# Comparative Effectiveness of Oral Endotracheal Tube Securement Methods in Adults: A Systematic Review and Network Meta-Analysis

**DOI:** 10.3390/nursrep16090317

**Published:** 2026-09-03

**Authors:** Chen-Jin Liao, Su-Er Guo, Ming-Ju Hsieh, Li-Fen Chao

**Affiliations:** 1 Department of General Medicine, Taipei City Hospital Zhongxing Branch; No. 145, Zhengzhou Rd., Datong Dist., Taipei 103212, Taiwan; b6928@tpech.gov.tw; 2 Doctoral Program, School of Nursing, College of Medicine, National Taiwan University, No.1, Sec. 1, Jen Ai Rd., Zhongzheng Dist., Taipei 100233, Taiwan; 3 Department of Nursing, College of Medicine, National Sun Yat-sen University No. 70, Lienhai Rd., Gushan Dist., Kaohsiung 804201, Taiwan; seguo@mail.nsysu.edu.tw; 4 Division of Thoracic and Cardiovascular Surgery, Department of Surgery, Chang Gung Memorial Hospital, No. 5, Fu-Hsing St., Guishan Dist., Taoyuan 333423, Taiwan; hsiehmj2@gmail.com; 5 School of Medicine, College of Medicine, Chang Gung University, No. 259, Wenhua 1st Rd., Guishan Dist., Taoyuan 333323, Taiwan; 6 Center for Health and Geriatric Care Research and Development, Department of Nursing, Chang Gung University of Science and Technology, No. 261, Wenhua 1st Rd., Guishan Dist., Taoyuan 333324, Taiwan

**Keywords:** oral endotracheal tube, securement methods, displacement, pressure injury, network meta-analysis, nursing

## Abstract

**Background/Objectives:** Securement of oral endotracheal tubes (ETTs) is a critical component of airway management in mechanically ventilated adults. Inadequate fixation may lead to tube displacement, and device-related pressure injuries. The aim of the study was to evaluate the effectiveness and safety of different oral ETT securement evidence in adults. **Methods:** A comprehensive search was conducted on seven English and Chinese databases (Cochrane Library, PubMed, CINAHL, Web of Science, EMBASE, Airiti Library and CNKI) to identify clinical trials evaluating the effectiveness and safety of different oral endotracheal tube securement methods. Methodological quality of the trials was assessed using the Cochrane Risk of Bias 2 (RoB 2.0) and the Joanna Briggs Institute (JBI) tools. A frequentist network meta-analysis was performed to estimate odds ratios with 95% CIs. The review protocol was registered with INPLASY (INPLASY202480089). **Results:** A total of 45 studies published between 1993 and 2022 (17 in English and 28 in Chinese) met the inclusion criteria. Compared with adhesive tape, ETT holders (OR 0.15, 95% CI 0.08–0.27) and twill (OR 0.25, 95% CI 0.11–0.59) significantly reduced tube displacement, with no difference between them. For pressure injury, ETT holders showed a significantly lower risk compared with adhesive tape (OR 0.33, 95% CI 0.15–0.70), while evidence for twill was inconclusive. No major inconsistency was detected for pressure injury, whereas inconsistency could not be assessed for tube displacement because the network contained no closed loops. **Conclusions:** Both twill and ETT holders were more effective than adhesive tape in reducing oral ETT displacement. ETT holders demonstrated more consistent benefits across outcomes and significantly reduced the risk of device-related pressure injury, suggesting they may be the preferred securement method for mechanically ventilated adults. Further high-quality head-to-head trials are needed to strengthen the current evidence.

## 1. Introduction

Endotracheal intubation and mechanical ventilation are essential interventions in critical ill adults to maintain airway patency and support vital functions. However, the presence of an endotracheal tube (ETT) may cause discomfort, agitation, and psychological stress, increasing the risk of tube displacement and unplanned extubation (UE). UE can lead to serious adverse events, including hypoxia, hemodynamic instability, respiratory failure, and cardiac arrest. In addition, prolonged ETT placement may result in device-related complications such as pressure injuries, oral mucosal damage, and ventilator-associated pneumonia [1,2,3].

Effective ETT securement is a fundamental nursing intervention to minimize these complications. Common securement methods include adhesive tape, twill tape, and commercially available ETT holders [4]. However, inadequate fixation or prolonged pressure from securement devices may itself contribute to tube displacement and device-related injuries. Therefore, identifying the most effective and safe securement method is essential for improving patient outcomes in critical care.

An early systematic review by Gardner [5] evaluated ETT fixation methods but was limited by the small number of studies and methodological weaknesses. Since then, numerous clinical studies and commercially available securement devices have been introduced. However, previous reviews have primarily relied on pairwise comparisons, while direct head-to-head comparisons across all available securement methods remain limited. Consequently, the comparative effectiveness and relative ranking of adhesive tape, twill tape, and commercially available ETT holders remain uncertain.

Given the limited availability of head-to-head comparisons among all securement methods, a network meta-analysis (NMA) may provide a more comprehensive comparison of the available interventions.

This study aimed to systematically synthesize and compare the effectiveness of oral endotracheal tube securement methods in adult patients through a network meta-analysis to inform clinical practice. Accordingly, this study was designed to address the following research questions:

**RQ1.**  *What types of oral endotracheal tube securement methods have been evaluated in adult patients undergoing mechanical ventilation?*

**RQ2.**  *What is the comparative effectiveness of different securement methods in preventing tube displacement and unplanned extubation?*

**RQ3.**  *How do different securement methods compare in terms of device-related complications, particularly facial or oral pressure injuries?*

**RQ4.**  *Based on a network meta-analytic framework, how can the available evidence be synthesized to determine the relative ranking and clinical performance of different securement methods?*

## 2. Methods

This study was conducted as a systematic review with a planned network meta-analysis (NMA). The review protocol was developed a priori and registered in the International Platform of Registered Systematic Review and Meta-Analysis Protocols (INPLASY; registration number: INPLASY202480089; registration date: 19 August 2024). The conduct and reporting of this study followed the Preferred Reporting Items for Systematic Reviews and Meta-Analyses extension guidelines for network meta-analysis (PRISMA NMA) [6]. The research question was formulated using the PICO framework: P (population), adult patients undergoing oral endotracheal intubation; I (intervention), ETT securement methods; C (comparison), different securement methods; and O (outcomes), tube displacement, unplanned extubation, and device-related complications (e.g., pressure or mucosal injuries).

### 2.1. Search Strategy

A comprehensive literature search was independently conducted by two investigators (CJL and LFC) across the following databases—Cochrane Library, PubMed, CINAHL, Web of Science, EMBASE, Airiti Library and the China National Knowledge Infrastructure (CNKI)—from inception to June 2026. Search terms included combinations of keywords and MeSH terms related to “endotracheal” and “securement” (e.g., fixation, twill, adhesive tape, endotracheal tube holder). Reference lists of included studies and relevant reviews were also screened. No language restrictions were applied.

### 2.2. Inclusion and Exclusion Criteria

Studies were included if they: (1) involved adults (≥18 years) undergoing oral endotracheal intubation; (2) evaluated at least one ETT securement (e.g., adhesive tape, twill, or ETT holders); (3) reported outcome related to securement, such as tube displacement, unplanned extubation, or device-related complications (e.g., facial pressure or mucosal injuries); and (4) used a comparative study design, including randomized controlled trials (RCTs), quasi-experimental studies, or observational studies with a comparison group.

Studies were excluded if they involved pediatric populations, nasotracheal intubation, tracheostomy tubes, or simulation settings, or were case reports, reviews, conference abstracts without full text, or non-comparative studies.

### 2.3. Methodological Quality Assessment

Two researchers independently assessed the risk of bias. RCTs were evaluated using the Cochrane Risk of Bias tool, version 2 (RoB 2) [7], and quasi-experimental studies were assessed using the Joanna Briggs Institute (JBI) critical appraisal checklist. For descriptive purposes, studies were categorized according to the proportion of applicable JBI appraisal criteria met—≥80% as high quality, 50–79% as moderate quality, and <50% as low quality—based on thresholds used in a previous systematic review [8]. Disagreements were resolved through discussion or consultation with a third reviewer.

### 2.4. Data Extraction and Management

Data were independently extracted by two researchers (CJL and LFC) using a standardized data extraction form. Discrepancies were resolved by discussion or consultation with a third researcher. Extracted data included study characteristics, participant characteristics, ETT securement methods, outcomes, and main findings. For each clinical outcome, the number of participants experiencing the event and the total number of participants in each intervention group were extracted to calculate odds ratios and corresponding 95% confidence intervals.

### 2.5. Statistical Analyses

A network meta-analysis was conducted to synthesize direct and indirect evidence across the three securement methods and estimate their relative treatment effects and rankings. Odds ratios (ORs) with 95% confidence intervals (CIs) were calculated for dichotomous outcomes. Analyses were performed using MetaInsight software (version 4.0.2, NIHR Complex Reviews Support Unit, London, UK) within a frequentist framework [9]. A network plot was generated to illustrate the geometry of the available comparisons among different ETT securement methods. Additional network analyses were conducted using the *netmeta* package in R (version 4.5.2; R Foundation for Statistical Computing, Vienna, Austria), including the assessment of network heterogeneity and treatment ranking. Network heterogeneity was quantified using the I^2^ statistic and between-study variance (τ^2^). Inconsistency between direct and indirect evidence was assessed where closed loops were available, with *p* < 0.05 indicating statistically significant inconsistency. Treatment rankings were based on P-scores, with higher values indicating more favorable treatment rankings. Statistical significance was defined as a two-tailed *p* value of less than 0.05.

### 2.6. Sensitivity Analyses

Sensitivity analyses were conducted using a leave-one-out approach to evaluate the robustness of the pooled estimates. In this procedure, each study was sequentially excluded, and the meta-analysis was repeated to examine the influence of individual studies on the overall effect estimates and treatment rankings. All sensitivity analyses were performed using the *netmeta* package in R (version 4.5.2; R Foundation for Statistical Computing, Vienna, Austria).

### 2.7. Publication Bias Assessment

Publication bias was assessed using funnel plots and Egger’s test when applicable. Funnel plot asymmetry was visually examined.

## 3. Results

**Figure 1:**
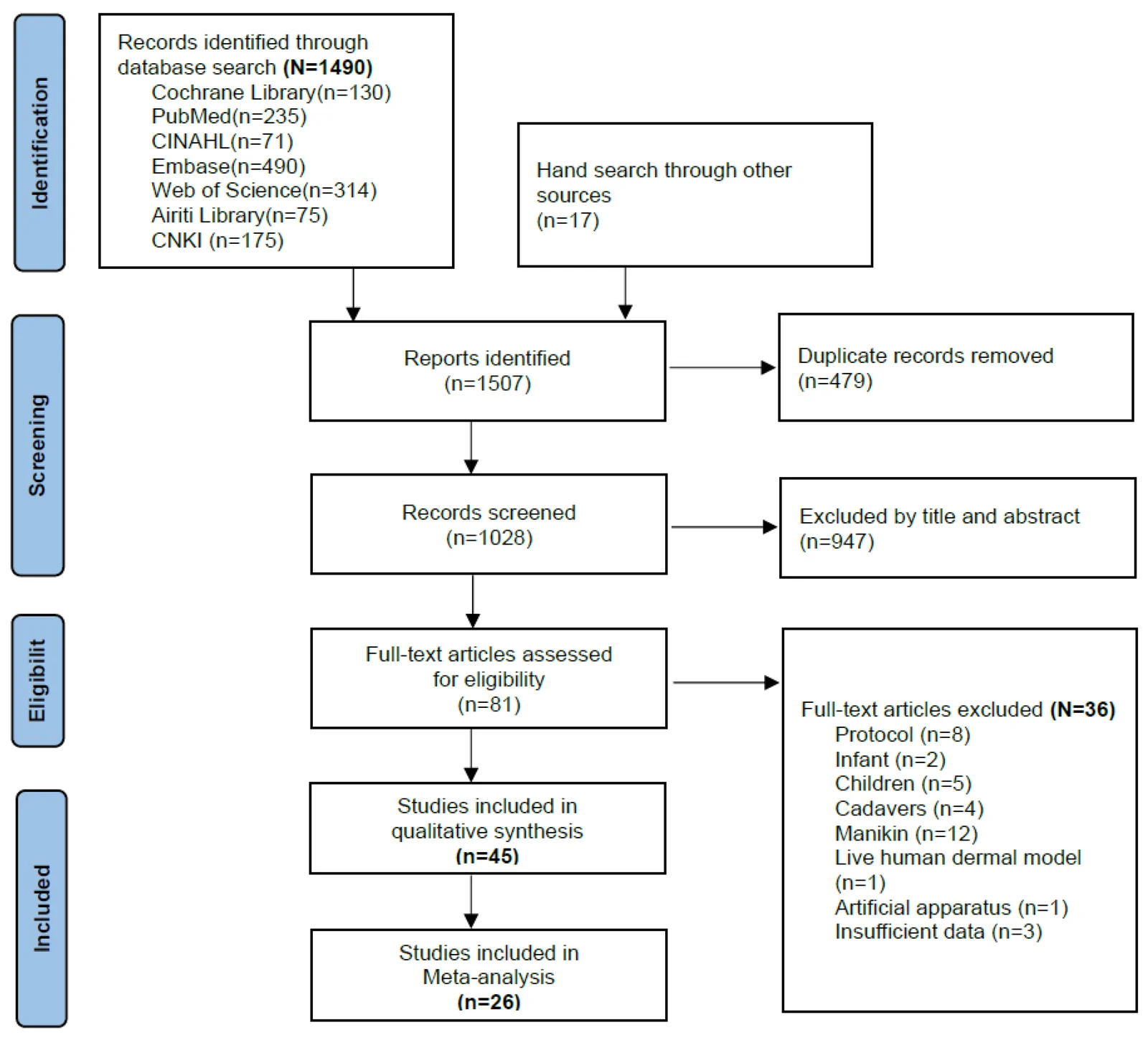
Flow diagram for the study selection process.

A total of 1507 records were identified from databases and reference lists. After screening titles and abstracts, 81 studies were assessed for eligibility. Of these, 36 were excluded due to ineligible design (n = 8), population (n = 25), or insufficient outcome data (n = 3). Ultimately, 45 studies were included in the systematic review, 26 provided sufficient data and were included in the quantitative synthesis. The study selection process is shown in Figure 1. The PRISMA-NMA checklist and database search details are provided in Tables S1 and S2.

### 3.1. Characteristics of Included Studies

All studies were conducted predominantly in critical care settings, with some studies conducted in perioperative settings. Of the 45 studies [10,11,12,13,14,15,16,17,18,19,20,21,22,23,24,25,26,27,28,29,30,31,32,33,34,35,36,37,38,39,40,41,42,43,44,45,46,47,48,49,50,51,52,53,54], eight were randomized controlled trials [10,11,12,13,14,15,16,17], whereas the remaining 37 were quasi-experimental studies; 17 were written in English (38%) and 28 in Chinese (62%). Studies were published between 1993 and 2022 and were conducted across 10 countries, primarily China and the United States. The mean age of patients ranged from 20 to 86 years.

Reported outcomes mainly included ETT-related events and device-related complications, such as tube displacement, unplanned extubation, mucosal and skin injuries, oral hygiene, gingival condition, cardiopulmonary physiological parameters, cost-effectiveness, and patient satisfaction. A brief summary of the characteristics of the included studies is presented in Table 1. The distribution of study characteristics across ETT securement comparisons is presented in Table 2, with detailed data provided in Table S3.

### 3.2. Quality Assessment

Risk of bias was assessed according to study design. Among the 8 RCTs, most were judged as having some concerns (75%, 6/8), and the remainder as having a high risk of bias (25%, 2/8); none were rated as low risk. Details are presented in Table S4 and Figure S1.

The 37 non-randomized controlled studies were assessed using the JBI checklist, with 6 rated as high-quality and 31 as moderate-quality; none were classified as low-quality. Detailed results are provided in Table S5.

### 3.3. Clinical Outcome: Displacement

**Figure 2:**
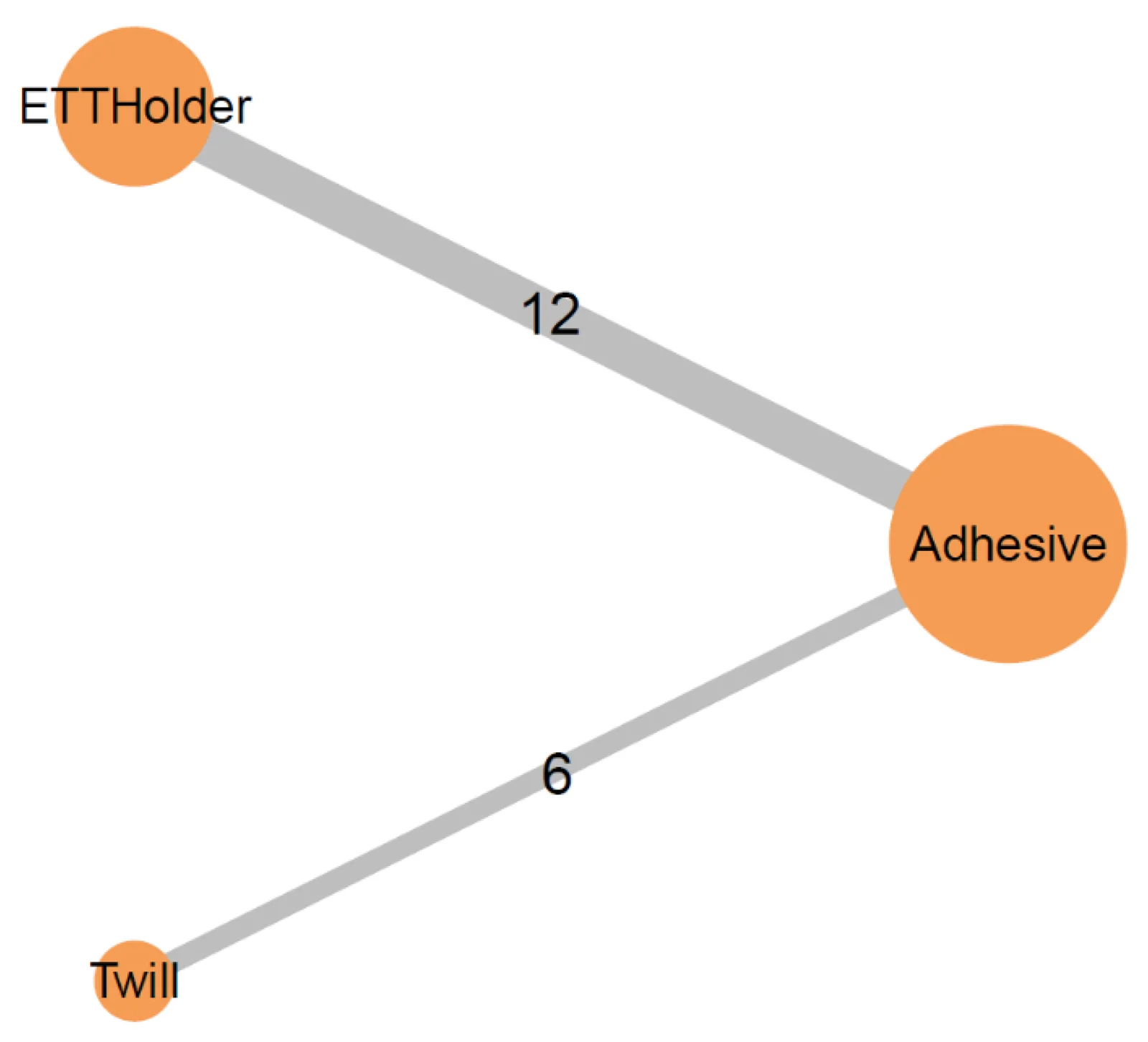
Network plot of endotracheal tube securement methods for tube displacement. The size of each node is proportional to the number of participants receiving each securement method, and the thickness of each edge is proportional to the number of studies directly comparing the connected methods. Numbers on the edges indicate the number of studies contributing to each direct comparison.

Eighteen studies were included in the network meta-analysis of tube displacement. Three securement methods were evaluated. Direct comparisons were available between adhesive tape and twill, as well as between adhesive tape and ETT holders, while no direct comparison between twill and ETT holders was identified. The network plot is shown in Figure 2.

**Figure 3:**
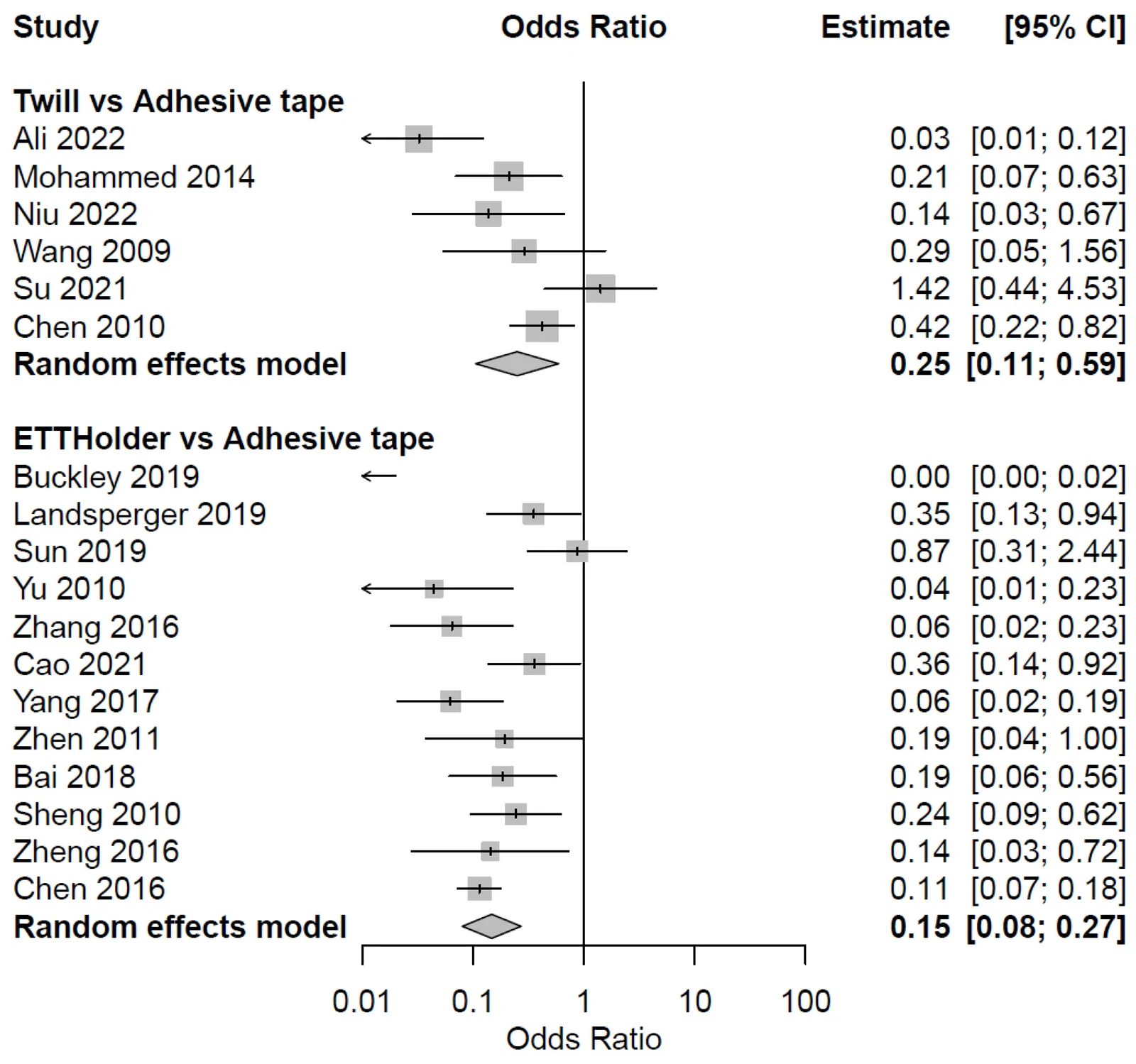
Forest plot of pairwise comparisons between endotracheal tube securement methods for tube displacement [11,14,17,23,26,28,29,30,31,34,36,40,41,42,47,48,53,54].

Compared with adhesive tape, twill generally showed a lower risk of tube-related complications, although some estimates were imprecise. ETT holders consistently demonstrated a lower risk, with most comparisons reaching statistical significance. Overall, the findings favored ETT holders over adhesive tape (Figure 3).

In the pairwise meta-analysis, the pooled estimates showed that both ETT holders (OR 0.15, 95% CI 0.08–0.27) and twill (OR 0.25, 95% CI 0.11–0.59) significantly reduced tube displacement compared with adhesive tape. In the network meta-analysis, the findings for ETT holders and twill versus adhesive tape were consistent with the pairwise estimates, with both methods showing significantly lower risks of tube displacement. The indirect comparison between ETT holders and twill favored ETT holders (OR 0.59, 95% CI 0.20–1.70), although the difference was not statistically significant. Based on P-score ranking, ETT holders ranked highest for tube displacement (0.918), followed by Twill (0.582) and adhesive tape (<0.001). Detailed comparisons and rankings are presented in Table 3.

### 3.4. Clinical Outcome: Pressure Injury

**Figure 4:**
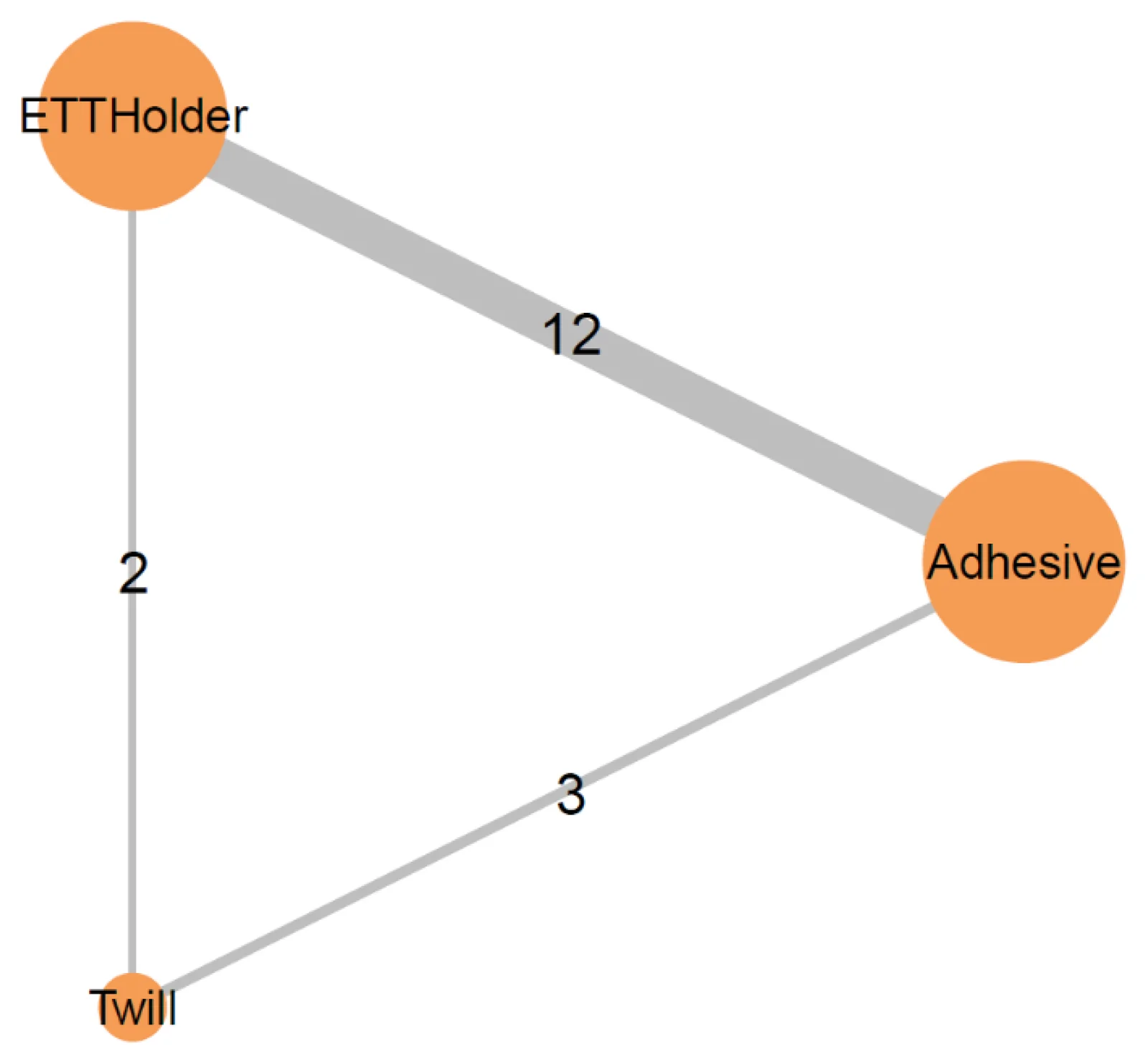
Network plot of endotracheal tube securement methods for pressure injury. The size of each node is proportional to the number of participants receiving each securement method, and the thickness of each edge is proportional to the number of studies directly comparing the connected methods. Numbers on the edges indicate the number of studies contributing to each direct comparison.

Seventeen studies were included in the network meta-analysis of pressure injury. Three securement methods were evaluated, with direct comparisons available among all interventions. The network plot is shown in Figure 4.

**Figure 5:**
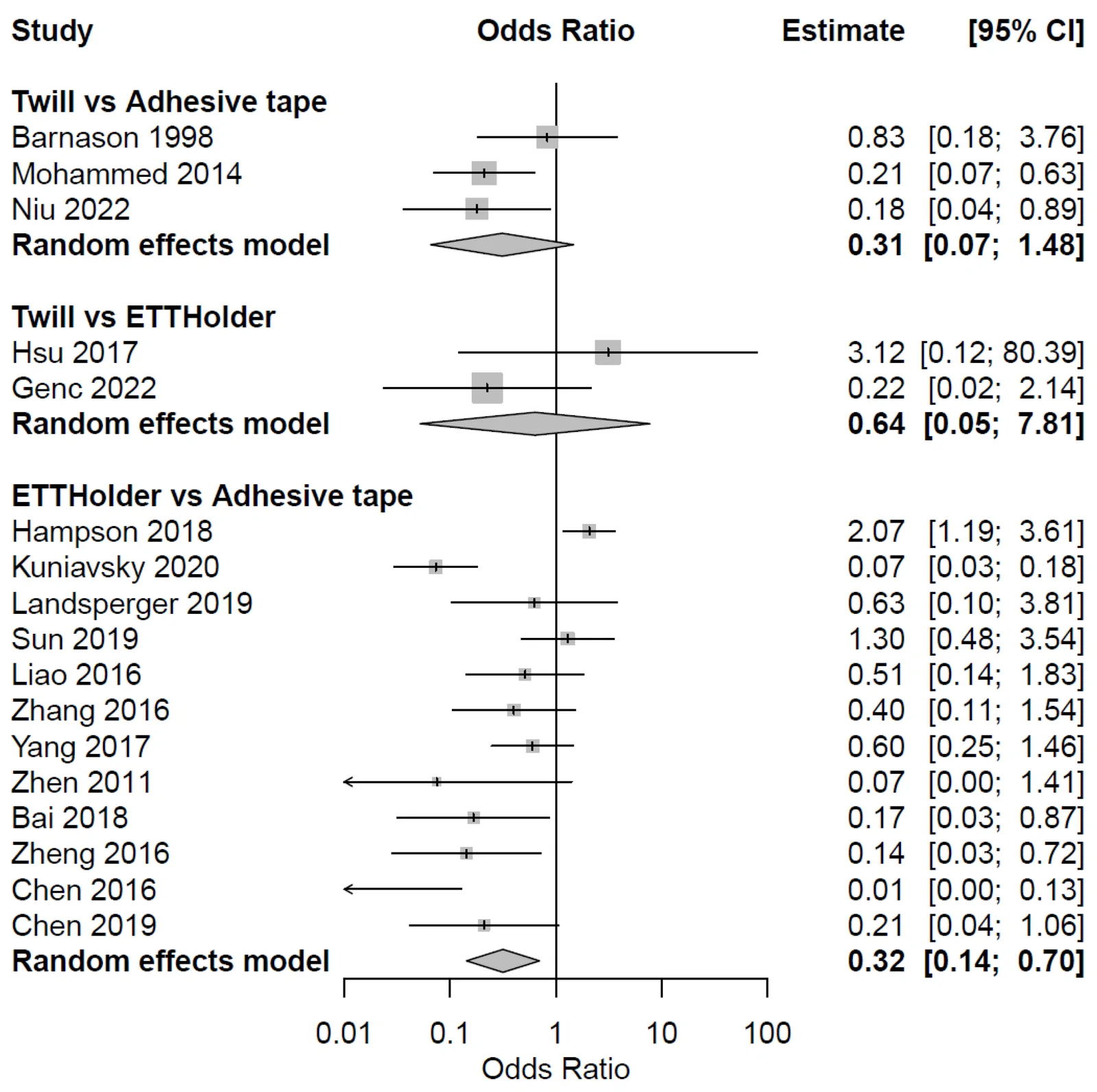
Forest plot of pairwise comparisons between different endotracheal tube securement methods for pressure injury [10,13,14,16,19,22,23,25,27,34,40,41,42,45,47,48,50].

Compared with adhesive tape, twill showed a trend toward reduced pressure injury, although results were inconsistent. ETT holders demonstrated a more consistent reduction in pressure injury compared with adhesive tape, with several comparisons reaching statistical significance. Comparisons between twill and ETT holders were inconclusive due to imprecise estimates. Overall, the findings favored ETT holders for reducing pressure injury (Figure 5).

For pressure injury, the pairwise meta-analysis showed that the pooled estimate significantly favored ETT holders over adhesive tape (OR 0.32, 95% CI 0.14–0.70). In the network meta-analysis, no significant difference was observed between twill and adhesive tape (OR 0.28, 95% CI 0.07–1.06) or between twill and ETT holders (OR 0.85, 95% CI 0.20–3.58). In contrast, ETT holders significantly reduced the risk of pressure injury compared with adhesive tape (OR 0.33, 95% CI 0.15–0.70). Based on P-score ranking, twill ranked highest for pressure injury (0.778), followed by ETT holders (0.706) and adhesive tape (0.016). However, the difference between twill and ETT holders was not statistically significant. Detailed results are presented in Table 4.

### 3.5. Heterogeneity and Inconsistency Test

Substantial heterogeneity was observed across the network for tube displacement (I^2^ = 72.1%, τ^2^ = 0.77) and pressure injury (I^2^ = 76.9%, τ^2^ = 1.39). Inconsistency could not be assessed for tube displacement because of the absence of closed loops in the network. For pressure injury, no significant inconsistency was identified between direct and indirect evidence (all *p* > 0.05). Detailed results are presented in Tables S6 and S7.

### 3.6. Sensitivity Analyses

Leave-one-out analyses showed that pooled estimates for tube displacement and pressure injury remained stable after sequential exclusion of individual studies, indicating robust findings (Figures S2 and S3).

### 3.7. Publication Bias

Publication bias was assessed using funnel plots and Egger’s test. Funnel plots appeared symmetrical for tube displacement, with no evidence of small-study effects (Egger’s test *p* = 0.611) (Figure S4). For pressure injury, funnel plot asymmetry was observed, and Egger’s test indicated potential small-study effects (*p* = 0.017) (Figure S5). These findings should be interpreted with caution due to the limited number of studies.

## 4. Discussion

### 4.1. Main Findings and Clinical Implications

Endotracheal intubation is a fundamental intervention in critical care, and effective ETT securement is essential for maintaining airway stability and preventing device-related complications. Using a network meta-analysis framework, this study synthesized direct and indirect evidence to compare commonly used securement methods.

The findings indicate that both twill and ETT holders reduce tube displacement compared with adhesive tape, with ETT holders demonstrating the most consistent benefit. For pressure injury, ETT holders showed a significant advantage over adhesive tape, whereas evidence for twill remained inconclusive. However, several individual studies, particularly those comparing adhesive tape with ETT holders, showed large effect estimates with wide confidence intervals, reflecting imprecision in individual study estimates. Nevertheless, leave-one-out sensitivity analyses showed that the sequential exclusion of individual studies did not materially alter the overall findings or treatment rankings.

From a clinical perspective, these results highlight the importance of securement strategies in reducing device-related complications among mechanically ventilated patients. Reducing tube displacement and pressure injury is critical, as these events may contribute to unplanned extubation and adverse outcomes. Based on the available evidence, ETT holders may be preferred in critical care settings due to their consistent performance across outcomes. However, no statistically significant difference in tube displacement was observed between ETT holders and twill. Twill may serve as a reasonable alternative when commercial devices are not available; however, its comparative effectiveness relative to ETT holders remains uncertain.

### 4.2. Interpretation of Results

Although ETT holders ranked highest in network meta-analysis, practical considerations may influence their implementation. Commercial holders are associated with higher costs and may not always conform optimally to patients with smaller facial structures, potentially leading to uneven pressure distribution. In such cases, supplementary padding may be required to reduce focal pressure, which may increase nursing workload and necessitate closer monitoring [26]. Twill fixation, in contrast, is inexpensive and widely available. However, its effectiveness is highly dependent on the skill of the operator; improper tying techniques may compromise stability, which remains a practical concern in clinical use.

Adhesive tape, although ranked last, continues to be widely used due to its simplicity and ease of application. Its fixation strength, however, is often affected by factors such as perspiration, skin oils, and friction. Notably, Chen [50] found that elastic adhesive tape outperformed infant-specific Transpore tape in clinical practice. Similarly, Li [55] evaluated five commonly used adhesive tapes (Transpore tape, Urgosyval tape, Transpore White tape, Multipore tape, and Durapore tape) using a manikin model and identified variations in their fixation performance. These findings suggest that material selection for adhesive tape may mitigate its disadvantages and minimize the risk of device-related skin injury in clinical practice.

The observed heterogeneity across studies likely reflects variability in patient characteristics, intervention implementation, and outcome definitions. In particular, the lack of a standardized definition for tube displacement represents a major methodological limitation in the field. Differences in whether minor positional shifts or clinically significant migration were reported may have influenced pooled estimates. Similarly, the assessment and reporting of pressure injury varied across studies, which may have reduced comparability. Furthermore, variations in device types, fixation techniques, and study design likely contributed to between-study variability. These sources of clinical and methodological heterogeneity should be considered when interpreting the pooled estimates.

### 4.3. Limitation

Several limitations should be considered. First, many included studies were small and at risk of bias, which may affect the robustness of the estimates. Second, substantial heterogeneity existed across studies, likely due to differences in patient populations, intervention techniques, and outcome definitions, particularly for tube displacement. Third, incomplete outcome reporting, such as limited information on pressure injury severity, restricted further analyses.

Fourth, although network meta-analysis enables indirect comparisons, the lack of high-quality head-to-head trials means that some estimates relied on indirect evidence. In addition, inconsistency could not be assessed for tube displacement because the network contained no closed loops, precluding an evaluation of agreement between direct and indirect evidence for this outcome. Fifth, a substantial proportion of the included studies were conducted in China, which may limit the generalizability of the findings to other geographical regions and healthcare settings. Sixth, the included studies spanned approximately three decades, during which ETT securement materials, device designs, and clinical practices may have evolved, potentially contributing to clinical heterogeneity. Finally, the assessment of publication bias was limited by the number of studies. Although Egger’s test suggested potential small-study effects for pressure injury, this may also reflect heterogeneity and methodological variability. Therefore, the findings for pressure injury should be interpreted with caution.

## 5. Conclusions

This systematic review and network meta-analysis provides comprehensive evidence on the comparative effectiveness of endotracheal tube securement methods. Both twill and ETT holders reduced tube displacement compared with adhesive tape, with ETT holders showing the most consistent benefit. ETT holders were also associated with a lower risk of pressure injury, However, comparative evidence involving twill, particularly against ETT holders, remains limited and imprecise. Further high-quality, adequately powered head-to-head trials are needed to clarify the comparative effectiveness of these securement methods.

These findings highlight the importance of securement strategies in reducing device-related complications in mechanically ventilated patients and support the use of ETT holders in critical care practice. Further high-quality, multicenter studies are needed to strengthen the evidence base and inform clinical guidelines.

## Figures and Tables

**Table 1:** Brief summary of study characteristics and outcomes.

	First Author (Year)	Country	Study Design	Sample Size	Comparison	Outcome
1	Clarke (1998) [20]	Australia	Quasi	222	Twill vs. Twill	Displacement, UE, Movement, PI, Nurse satisfaction
2	Lee (2016) [43]	China	Quasi	100	Twill vs. Twill	Displacement, Patient satisfaction
3	Levy (1993) [18]	USA	Quasi	136	Twill vs. Adhesive	Movement, Oral hygiene, Patient satisfaction, Nurse satisfaction
4	Barnason (1998) [19]	USA	Quasi	52	Twill vs. Adhesive	UE, PI, Oral mucosa injury
5	Wang (2009) [28]	China	Quasi	64	Twill vs. Adhesive	Displacement, UE
6	Chen (2010) [30]	China	Quasi	212	Twill vs. Adhesive	Displacement
7	Hsieh (2010) [32]	China	Quasi	200	Twill vs. Adhesive	Displacement, UE
8	Seyedhosseini (2017) [21]	Iran	Quasi	72	Twill vs. Adhesive	UE
9	Su (2021) [54]	China	Quasi	80	Twill vs. Adhesive	Displacement, Patient satisfaction
10	Niu (2023) [17]	China	RCT	85	Twill vs. Adhesive	Displacement, PI, Time, Cost
11	Ali (2022) [26]	Egypt	Quasi	74	Twill vs. Adhesive	Displacement, Oral mucosa injury
12	Hsu (2017) [45]	China	Quasi	50	Twill vs. ETT holders	Displacement, UE, PI
13	Hampson (2018) [22]	Australia	Quasi	2008	Twill vs. ETT holders	PI
14	Genc (2022) [16]	Turkey	RCT	60	Twill vs. ETT holders	PI
15	Liu (2011) [35]	China	Quasi	638	Adhesive vs. Adhesive	Displacement, UE, PI
16	Chen (2013) [36]	China	Quasi	100	Adhesive vs. Adhesive	Displacement, UE, Oral mucosa injury, Oral hygiene
17	Gou et al. (2015) [38]	China	Quasi	85	Adhesive vs. Adhesive	Displacement, UE
18	Zeng et al. (2016) [10]	Singapore	RCT	60	Adhesive vs. Adhesive	PI
19	Fang (2016) [39]	China	Quasi	86	Adhesive vs. Adhesive	Displacement, UE, Oral mucosa injury, Patient satisfaction
20	Chen (2017) [46]	China	Quasi	90	Adhesive vs. Adhesive	Displacement, PI, Patient satisfaction, Nurse satisfaction, Time
21	Chen (2020) [51]	China	Quasi	100	Adhesive vs. Adhesive	Displacement, PI, Oral mucosa injury, Oral hygiene, Patient satisfaction, Time
22	Xia (2020) [52]	China	Quasi	53	Adhesive vs. Adhesive	Displacement, Oral mucosa injury
23	Bahadori (2022) [15]	USA	RCT	141	Adhesive vs. Adhesive	Erythema, Oedema, Scaling, Tearing
24	Yu (2010) [29]	China	Quasi	40	Adhesive vs. ETT holders	Displacement, UE
25	Sheng (2010) [31]	China	Quasi	40	Adhesive vs. ETT holders	Displacement, Oral hygiene
26	Zhen, Xu (2011) [34]	China	Quasi	65	Adhesive vs. ETT holders	Displacement, UE, PI
27	Buckley (2016) [11]	USA	RCT	60	Adhesive vs. ETT holders	Displacement
28	Byun (2016) [12]	Korea	RCT	48	Adhesive vs. ETT holders	Displacement, Movement
29	Liao (2016) [27]	Taiwan	Quasi	97	Adhesive vs. ETT holders	PI, Time, Cost
30	Zhang (2016) [40]	China	Quasi	120	Adhesive vs. ETT holders	Displacement, Movement, PI, Oral mucosa injury, Nurse satisfaction, Time
31	Chen (2016) [41]	China	Quasi	36	Adhesive vs. ETT holders	Displacement, PI, Oral hygiene, Time, Cost
32	Zheng (2016) [42]	China	Quasi	60	Adhesive vs. ETT holders	Displacement, PI, VAP, Patient satisfaction
33	Yang (2017) [47]	China	Quasi	80	Adhesive vs. ETT holders	Displacement, Movement, PI, Nurse satisfaction
34	Bai et al. (2018) [48]	China	Quasi	60	Adhesive vs. ETT holders	Displacement, Movement, PI, Nurse satisfaction
35	Chen (2019) [50]	China	Quasi	78	Adhesive vs. ETT holders	Movement, PI, Oral mucosa injury, Nurse satisfaction, Time
36	Landsperger (2019) [13]	USA	RCT	298	Adhesive vs. ETT holders	Displacement, UE, PI, Oral mucosa injury, VAP, Mortality
37	Cao (2021) [53]	China	Quasi	87	Adhesive vs. ETT holders	Displacement, UE, Movement, PI, Oral mucosa injury, Time
38	Yanık (2022) [24]	Turkey	Quasi	86	Adhesive vs. ETT holders	SBP, DBP, HR, SpO2
39	Kuniavsky (2020) [25]	Israel	Quasi	155	ETT holders vs. ETT holders	PI, Hospital days, Ventilation days
40	He (2011) [33]	China	Quasi	90	Triple comparison	Displacement, UE
41	Mohammed (2015) [14]	Egypt	RCT	90	Triple comparison	Displacement, PI, Oral mucosa injury, Time, EJVP, Pain, Patient satisfaction
42	Liang (2015) [37]	China	Quasi	90	Triple comparison	UE, Complication Events
43	Hsieh (2017) [44]	China	Quasi	120	Triple comparison	Displacement, Oral mucosa injury, Oral hygiene, Patient satisfaction, Time
44	Sun (2019) [23]	China	Quasi	95	Triple comparison	Displacement, PI
45	Zhang (2019) [49]	China	Quasi	150	Triple comparison	Displacement, UE, PI, Oral hygiene, Time

**Table 2:** Distribution of study characteristics by ETT securement comparison.

Study Characteristics	Total	Comparison						
		T1 vs. T2	T vs. A	T vs. E	A1 vs. A2	A vs. E	E1 vs. E2	Three-Arm
Study design								
RCT	8	0	1	1	2	3	0	1
Quasi-experimental	37	2	8	2	7	12	1	5
Year								
Before 2000	3	1	2	0	0	0	0	0
2000–2010	6	0	3	0	0	2	0	1
2011–2020	29	1	1	2	8	11	1	5
2021 onward	7	0	3	1	1	2	0	0
Country								
United States	6	0	3	0	1	2	0	0
China	29	1	5	1	7	10	0	5
Other	10	1	1	2	1	3	1	1

Note: T, twill; A, adhesive tape; E, ETT holders. Comparisons within the same securement category (e.g., T1 vs. T2) represent comparisons of different materials or fixation techniques within that category. Three-arm refers to studies evaluating three securement intervention arms, irrespective of the specific combination of securement methods.

**Table 3:** League table of pairwise and network meta-analysis estimates for tube displacement.

ETT holders	-	0.15 [0.08; 0.27]
0.59 [0.20; 1.70]	Twill	0.25 [0.11; 0.59]
0.15 [0.08; 0.27]	0.25 [0.11; 0.59]	Adhesive tape

Note: Treatments are ranked from best to worst along the leading diagonal. Estimates above the leading diagonal are derived from pairwise meta-analyses and represent the treatment in the column versus the treatment in the row. Estimates below the leading diagonal are derived from network meta-analyses and represent the treatment in the row versus the treatment in the column.

**Table 4:** League table of pairwise and network meta-analysis estimates for pressure injury.

Twill	0.64 [0.05; 7.81]	0.31 [0.07; 1.48]
0.85 [0.20; 3.58]	ETT holders	0.32 [0.14; 0.70]
0.28 [0.07; 1.06]	0.33 [0.15; 0.70]	Adhesive tape

Note: Treatments are ranked from best to worst along the leading diagonal. Estimates above the leading diagonal are derived from pairwise meta-analyses and represent the treatment in the column versus the treatment in the row. Estimates below the leading diagonal are derived from network meta-analyses and represent the treatment in the row versus the treatment in the column.

## Data Availability

The original contributions presented in the study are included in the article/Supplementary Material, further inquiries can be directed to the corresponding author/s.

## Supplementary Materials

The following supporting information can be downloaded at: https://www.mdpi.com/article/10.3390/nursrep16090317/s1, Table S1: PRISMA for network meta-analysis checklist; Table S2: Keywords and search results in different databases; Table S3: Detailed characteristics and outcome data of the included studies; Table S4: Quality assessment of individual RCTs using Cochrane risk of bias 2 tool; Table S5: Quality assessment of quasi-experimental studies using the JBI appraisal tool; Table S6: Inconsistency test results for tube displacement in the network meta-analysis; Table S7: Inconsistency test results for pressure injury in the network meta-analysis; Figure S1: Summary of quality assessment of studies included in the network meta-analysis using Cochrane risk of bias 2 tool; Figure S2: Forest plots of leave-one-out sensitivity analysis for tube displacement; Figure S3: Forest plots of leave-one-out sensitivity analysis for pressure injury; Figure S4: Funnel plot: Adhesive tape vs. ETT holder in displacement event; Figure S5: Funnel plot: Adhesive tape vs. ETT holders in pressure injury event.

## Public Involvement Statement

No public involvement in any aspect of this research.

## Guidelines and Standards Statement

The conduct and reporting of this study followed the Preferred Reporting Items for Systematic Reviews and Meta-Analyses extension guidelines for network meta-analysis (PRISMA-NMA).

## Use of Artificial Intelligence

Artificial intelligence (AI)-assisted tools (ChatGPT, GPT-5.5, OpenAI) were used solely for language polishing to improve the clarity, grammar, and readability of the manuscript. No AI tools were used for the study design, literature selection, data extraction, data analysis, interpretation of the findings, or generation of scientific content. All AI-assisted suggestions were carefully reviewed, revised as appropriate, and approved by the authors, who take full responsibility for the content of the manuscript.

## Abbreviations

The following abbreviations are used in this manuscript:

CIs	Confidence intervals
CINAHL	Cumulative Index of Nursing and Allied Health Literature
CNKI	China National Knowledge Infrastructure
ETT	Endotracheal tube
JBI	Joanna Briggs Institute
MeSH	Medical Subject Headings
NMA	Network meta-analysis
ORs	Odds ratios
PI	Pressure injury
PRISMA	Preferred Reporting Items for Systematic reviews and Meta-Analyses
RCT	Randomized controlled trials
RoB 2.0	Cochrane Risk of Bias 2
UE	Unplanned extubation
VAP	Ventilator-associated pneumonia
