# Supplementary material for: Comparative Effectiveness of Oral Endotracheal Tube Securement Methods in Adults: A Systematic Review and Network Meta-Analysis
Source: Nurs Rep. 2026 Sep 3;16(9):317. doi: 10.3390/nursrep16090317 (PMC13609965; doi:10.3390/nursrep16090317)

Supplementary material

Table S1. *PRISMA for network meta-analysis checklist*

Section and Topic	#	Checklist item	Location
**Title**			
Title	1	Identify the report as a systematic review incorporating a network meta-analysis (or related form of meta-analysis).	Title
**Abstract**			
Structured summary	2	Provide a structured summary including, as applicable: **Background**: main objectives / **Methods**: data sources; study eligibility criteria, participants, and interventions; study appraisal; and synthesis methods, such as network meta-analysis. / **Results**: number of studies and participants identified; summary estimates with corresponding confidence/credible intervals; treatment rankings may also be discussed. Authors may choose to summarize pairwise comparisons against a chosen treatment included in their analyses for brevity. / **Discussion/conclusions**: limitations; conclusions and implications of findings. / **Other**: primary source of funding; systematic review registration number with registry name.	Abstract
**Introduction**			
Rationale	3	Describe the rationale for the review in the context of what is already known, including mention of why a network meta-analysis has been conducted.	Introduction
Objectives	4	Provide an explicit statement of questions being addressed, with reference to participants, interventions, comparisons, outcomes, and study design (PICOS).	Introduction
**Methods**			
Protocol & registration	5	Indicate whether a review protocol exists and where it can be accessed; and, if available, provide registration information, including registration number.	Methods/ 1st paragraph
Eligibility criteria	6	Specify study characteristics (e.g., PICOS, length of follow-up) and report characteristics (e.g., years considered, language, publication status) used as criteria for eligibility, giving rationale. Clearly describe eligible treatments included in the treatment network, and note whether any have been clustered or merged into the same node (with justification).	Methods/ 2.1 Search methods /2.2 Inclusion and exclusion criteria
Information sources	7	Describe all information sources (e.g., databases with dates of coverage, contact with study authors) in the search and date last searched.	Table S2
Search	8	Present full electronic search strategy for at least one database, including any limits used, such that it could be repeated.	Table S2
Study selection	9	State the process for selecting studies (i.e., screening, eligibility, included in systematic review, and, if applicable, included in the meta-analysis).	Methods/ 2.4 Data extraction and management Result/1st paragraph
Data collection	10	Describe method of data extraction from reports (e.g., piloted forms, independently, in duplicate) and any processes for obtaining and confirming data.	Methods/ 2.4 Data extraction and management
Data items	11	List and define all variables for which data were sought (e.g., PICOS, funding sources) and any assumptions and simplifications made.	Methods/ 2.4 Data extraction and management
Network geometry	S1	Describe methods used to explore the geometry of the treatment network under study and potential biases related to it. This should include how the evidence base has been graphically summarized for presentation, and what characteristics were compiled and used to describe the evidence base to readers.	Methods/ 2.5 Statistical Analyses
Risk of bias within	12	Describe methods used for assessing risk of bias of individual studies (including specification of whether this was done at the study or outcome level), and how this information is to be used in any data synthesis.	Methods/ 2.3 Methodological Quality appraisal
Summary measures	13	State the principal summary measures (e.g., risk ratio, difference in means). Also describe the use of additional summary measures assessed, such as treatment rankings, as well as modified approaches used to present summary findings from meta-analyses.	Methods/ 2.5 Statistical Analyses
Planned methods of analysis	14	Describe the methods of handling data and combining results of studies for each network meta-analysis. This should include, but not be limited to: Handling of multi-arm trials; Selection of variance structure; Selection of prior distributions in Bayesian analyses; and Assessment of model fit.	Methods/ 2.5 Statistical Analyses

Assessment of inconsistency	S2	Describe the statistical methods used to evaluate the agreement of direct and indirect evidence in the treatment network(s) studied. Describe efforts taken to address its presence when found.	Methods/ 2.5 Statistical Analyses
Risk of bias across	15	Specify any assessment of risk of bias that may affect the cumulative evidence.	Methods/ 2.3 Quality appraisal
Additional analyses	16	Describe methods of additional analyses if done, indicating which were pre-specified. This may include, but not be limited to, the following: Sensitivity or subgroup analyses; Meta-regression analyses; Alternative formulations of the treatment network; and Use of alternative prior distributions for Bayesian analyses (if applicable).	Methods/ 2.6 Sensitivity Analyses / 2.7 Publication bias Assessment
**Results**			
Study selection	17	Give numbers of studies screened, assessed for eligibility, and included in the review, with reasons for exclusions at each stage, ideally with a flow diagram.	Result/1st paragraph Figure 1, Table S2
Network structure	S3	Provide a network graph of the included studies to enable visualization of the geometry of the treatment network.	Figure 2 & 4
Network geometry	S4	Provide a brief overview of characteristics of the treatment network. This may include commentary on the abundance of trials and randomized patients for the different interventions and pairwise comparisons in the network, gaps of evidence in the treatment network, and potential biases reflected by the network structure.	Result/ 3.3 & 3.4 Clinical outcome Figure 2 & 4
Study characteristics	18	For each study, present characteristics for which data were extracted (e.g., study size, PICOS, follow-up period) and provide the citations.	Result/ 3.1 Characteristics of included studies Table 1 & Table S3
Risk of bias within	19	Present data on risk of bias of each study and, if available, any outcome level assessment.	3.2. Quality assessment Table S4, Table S5 Figure S1
Results of individual studies	20	For all outcomes considered (benefits or harms), present, for each study: (1) simple summary data for each intervention group, and (2) effect estimates and confidence intervals. Modified approaches may be needed to deal with information from larger networks.	Table S3
Synthesis of results	21	Present results of each meta-analysis done, including confidence/credible intervals. In larger networks, authors may focus on comparisons versus a particular comparator (e.g. placebo or standard care), with full findings presented in an appendix. League tables and forest plots may be considered to summarize pairwise comparisons. If additional summary measures were explored (such as treatment rankings), these should also be presented.	3.3 & 3.4 Clinical outcome Figure 3 & 5, Table 2 & 3
Exploration for inconsistency	S5	Describe results from investigations of inconsistency. This may include such information as measures of model fit to compare consistency and inconsistency models, P values from statistical tests, or summary of inconsistency estimates from different parts of the treatment network.	3.5 Heterogeneity and Inconsistency Test Table S6. S7
Risk of bias across	22	Present results of any assessment of risk of bias across studies for the evidence base being studied.	3.7 Publication bias Figure S4, S5
Additional analyses	23	Give results of additional analyses, if done (e.g., sensitivity or subgroup analyses, meta-regression analyses, alternative network geometries studied, alternative choice of prior distributions for Bayesian analyses, and so forth).	3.6 Sensitivity Analyses Figure S2, S3
**Discussion**			
Summary of evidence	24	Summarize the main findings, including the strength of evidence for each main outcome; consider their relevance to key groups.	Discussion
Limitations	25	Discuss limitations at study and outcome level (e.g., risk of bias), and at review level (e.g., incomplete retrieval of identified research, reporting bias). Comment on the validity of the assumptions, such as transitivity and consistency. Comment on any concerns regarding network geometry (e.g., avoidance of certain comparisons).	Discussion / 4.3 Limitations
Conclusions	26	Provide a general interpretation of the results in the context of other evidence, and implications for future research.	Conclusion
**Funding**			
Funding	27	Describe sources of funding for the systematic review and other support (e.g., supply of data); role of funders for the systematic review. This should also include information regarding whether funding has been received from manufacturers of treatments in the network and/or whether some of the authors are content experts with professional conflicts of interest that could affect use of treatments in the network.	Funding

Table S2. *Keywords and search results in different databases*

Database	Keyword	Filter	Date	Results
PubMed	("intubation intratracheal" OR "Intubated" OR "Endotracheal" OR "endotracheal tube" OR "intratracheal intubation" OR "intratracheal intubations" OR "endotracheal intubation" OR "endotracheal intubations") AND ("Twill" OR "twill tape" OR "cotton tape" OR "gauze roll" OR "Bandage" OR "non adhesive tape" OR "adhesive tape" OR ("endotracheal tube holder" OR "endotracheal tube fastener") OR "Suture")	Title Abstract	Jun. 12, 2026	235
Cochrane Reviews	("intubation intratracheal" OR "Intubated" OR "Endotracheal" OR "endotracheal tube" OR "intratracheal intubation" OR "intratracheal intubations" OR "endotracheal intubation" OR "endotracheal intubations") AND ("Twill" OR "twill tape" OR "cotton tape" OR "gauze roll" OR "Bandage" OR "non adhesive tape" OR "adhesive tape" OR ("endotracheal tube holder" OR "endotracheal tube fastener") OR "Suture")	Title Abstract	Jun. 12, 2026	20
Cochrane CENTRAL	("intubation intratracheal" OR "Intubated" OR "Endotracheal" OR "endotracheal tube" OR "intratracheal intubation" OR "intratracheal intubations" OR "endotracheal intubation" OR "endotracheal intubations") AND ("Twill" OR "twill tape" OR "cotton tape" OR "gauze roll" OR "Bandage" OR "non adhesive tape" OR "adhesive tape" OR ("endotracheal tube holder" OR "endotracheal tube fastener") OR "Suture")	Title Abstract	Jun. 12, 2026	110
Web of Science	("intubation intratracheal" OR "Intubated" OR "Endotracheal" OR "endotracheal tube" OR "intratracheal intubation" OR "intratracheal intubations" OR "endotracheal intubation" OR "endotracheal intubations") AND ("Twill" OR "twill tape" OR "cotton tape" OR "gauze roll" OR "Bandage" OR "non adhesive tape" OR "adhesive tape" OR ("endotracheal tube holder" OR "endotracheal tube fastener") OR "Suture")	Title Abstract	Jun. 12, 2026	314
CINAHL	("intubation intratracheal" OR "Intubated" OR "Endotracheal" OR "endotracheal tube" OR "intratracheal intubation" OR "intratracheal intubations" OR "endotracheal intubation" OR "endotracheal intubations") AND ("Twill" OR "twill tape" OR "cotton tape" OR "gauze roll" OR "Bandage" OR "non adhesive tape" OR "adhesive tape" OR ("endotracheal tube holder" OR "endotracheal tube fastener") OR "Suture")	Title Abstract	Jun. 12, 2026	71
EMBASE	("intubation intratracheal" OR "Intubated" OR "Endotracheal" OR "endotracheal tube" OR "intratracheal intubation" OR "intratracheal intubations" OR "endotracheal intubation" OR "endotracheal intubations") AND ("Twill" OR "twill tape" OR "cotton tape" OR "gauze roll" OR "Bandage" OR "non adhesive tape" OR "adhesive tape" OR ("endotracheal tube holder" OR "endotracheal tube fastener") OR "Suture")	Title Abstract	Jun. 12, 2026	490
Airiti Library (CEPS)	((氣管內管) OR (插管)) = 篇名.關鍵字.摘要 AND ((膠帶) OR (宜拉膠) OR (棉繩) OR (黏性膠帶) OR (氣管內管固定器) OR (縫線)) = 篇名.關鍵字.摘要	Title Abstract Keyword	Jun. 12, 2026	75

CNKI	(((题名或关键词:(氣管內管)) or (摘要:(氣管內管))) or ((题名或关键词:(插管)) or (摘要:(插管)))) AND (((题名或关键词:(棉繩)) or (摘要:(棉繩))) or ((题名或关键词:(膠帶)) or (摘要:(膠帶))) or ((题名或关键词:(宜拉膠)) or (摘要:(宜拉膠))) or ((题名或关键词:(黏性膠帶)) or (摘要:(黏性膠帶))) or ((题名或关键词:(氣管內管固定器)) or (摘要:(氣管內管固定器))) or ((题名或关键词:(縫線)) or (摘要:(縫線))))	Title Abstract	Jun. 12, 2026	175

Table S3. *Detailed characteristics and outcome data of the included studies*

	Author(yr)/Country<br>Design	Sample Characters	Intervention	Outcome measure	Measurement/time	Comparison Between Groups
**Twill vs Twill**						
1.	Clarke (1998)/Australia<br>Quasi	● ICU<br>● Age: 55.25±15.85	● Twill with knot (n=113)<br>● Twill with bow (n=109)	(1) Displacement<br>(2) UE<br>(3) Movement (cm)<br>(4) Pressure injury<br>(5) Nurse satisfaction (Likert)	QD	All N/R p-value<br>(1) knot (4%) vs bow (4%)<br>(2) knot (2%) vs bow (2%)<br>(3) -<br>(4) knot (12%) vs bow (9%)<br>(5) -
2.	Lee (2016)/China<br>Quasi	● ICU<br>● Age: 18-60	● Twill*3 (n=50)<br>● Twill*1 (n=50)	(1) Displacement<br>(2) Patient satisfaction<br>(Face rating scale)	N/R	(1) Twill*3 (8%) vs Twill*1 (40%), p<0.001<br>(2) Twill*3 (20%) vs Twill*1 (50%), p=0.002
**Twill vs Adhesive tape**						
3.	Levy, Griego (1993)/USA<br>Quasi	● ICU<br>● Age: 56.4±17.8	● Twill (n=34)<br>● Adhesive tape (n=33)<br>● TFXB (n=35)<br>● VFXB (n=34)	(1) Movement (cm)<br>(2) Oral hygiene (Likert)<br>(3) Patient satisfaction (Likert)<br>(4) Nurse satisfaction (Likert)	Twice daily	(2) A>TFXB(p<0.01)<br>A>VFXB(p<0.001)<br>(3) A>T(p<0.05), TFXB, VFXB(p<0.001)<br>(4) A>T, TFXB, VFXB(p<0.001);<br>T> TFXB(p<0.01), VFXB(p<0.001)<br>(5) A>T(p<0.05), TFXB, VFXB(p<0.001);<br>T> TFXB, VFXB(p<0.001)
4.	Barnason et al.(1998)/USA<br>Quasi	● ICU<br>● Age: 22-85	● Twill (n=28)<br>● Adhesive tape (n=24)	(1) UE<br>(2) Pressure injury<br>(3) Oral mucosa injury(OAG scale)	Twice daily	(1) Twill (14.2%) vs Adhesive (25%), p=0.45<br>(2) Twill (14.2%) vs Adhesive (16.7%)
5.	Wang et. al (2009)/ China<br>Quasi	● ICU<br>● Age: 42±6.8	● Twill (n=32)<br>● Adhesive tape (n=32)	(1) Displacement<br>(2) UE	N/R	(1) Twill (6.2%) vs Adhesive (18.8%), N/R p-value<br>(2) Twill (3.1%) vs Adhesive (9.3%), N/R p-value
6.	Chen et al. (2010)/China<br>Quasi	● ICU<br>● Age: 42-86	● Twill with tegaderm (n=108)<br>● Adhesive tape (n=104)	(1) Displacement	N/R	(1) Twill with tegaderm (15.7%) vs Adhesive (30.8%), p<0.001
7.	Hsieh (2010)/China<br>Quasi	● ICU<br>● Age: 45±3.1	● Twill (n=100)<br>● Adhesive tape (n=100)	(1) Displacement<br>(2) UE	N/R	(1) Twill (2%) vs Adhesive (17%), p<0.01<br>(2) Twill (0%) vs Adhesive (2%), p<0.01
8.	Seyedhosseini et al. (2017)/Iran<br>Quasi	● ED<br>● Age: 55.98±18.39	● Twill (n=36)<br>● Adhesive tape (n=36)	UE		(1) Twill (5.6%) vs Adhesive (16.7%), p=0.24
9.	Su (2021)/China<br>Quasi	● ICU<br>● Age: 60.7±5.96	● Twill (n=40)<br>● Adhesive tape (n=40)	(1) Displacement<br>(2) Patient satisfaction	N/R	(1) Twill (40%) vs Adhesive (30%), p=0.034<br>(2) Twill (37.5%) vs Adhesive (7.5%)
10.	Niu et. al (2023)/China	● ICU	● Twill(n=43)	(1) Displacement	Twice daily	(1) Twill (4.7%) vs Adhesive (26.2%), p<0.05

	Author(yr)/Country Design	Sample Characters	Intervention	Outcome measure	Measurement/time	Comparison Between Groups
	RCT	● Age: 61.24±6.71	● Adhesive tape (n=42)	(2) Pressure injury (NPIAP tool)<br>(3) Time<br>(4) Cost		(2) Twill (4.7%) vs Adhesive (21.4%), p<0.05<br>(3) Twill (5.16±0.79) vs Adhesive (6.67±1.074), p<0.05<br>(4) Twill (13.38±0.54) vs Adhesive (18.78±0.78), p<0.05
11.	Ali et. al (2022)/Egypt<br>Quasi	● SICU<br>● Age: 50.6±16.75	● Twill (n=37)<br>● Adhesive tape (n=37)	(1) Displacement (ETT slippage scale)<br>(2) Oral mucosa injury(OAG scale)	(1) 15, 30, 60, 120 mins<br>(2) 2, 6, 12, 24 hrs	(1) p= N/R , 0.011, <0.001, <0.001<br>(2) p= N/R , 1, <0.002, <0.001
**Twill vs ETT holder**						
12.	Hsu (2017)/ China<br>Quasi	● ICU<br>● Age: 55.14±1.11	● ETT holder (n=25)<br>● Twill (n=25)	(1) Displacement<br>(2) UE<br>(3) Pressure injury		(1) holder (4%) vs Twill (8%), p<0.05<br>(2) holder (8%) vs Twill (12%), p<0.05<br>(3) holder (0%) vs Twill (4%), p<0.05
13.	Hampson (2018)/Australia<br>Quasi	● ICU<br>● Age: 47.7-72.6	● Twill (n=1412)<br>● ETT holder (n=596)	Pressure injury(NPIAP tool)		holder (4%) vs Twill (2%), N/R p
14.	Genc, Yildiz (2022)/Turkey<br>RCT	● ICU<br>● Age: 51.03±10.29	● ETT holder (n=30)<br>● Twill (n=30)	(1) Pressure injury(NPIAP tool)<br>(2) Braden pressure ulcer risk measures<br>(3) Pressure ulcer scale for healing results (scale)<br>(4) Tissue surface area (event)<br>(5) Tissue type (event)	ETT reposition Q4H	(1) Twill (86.7%) vs ETT holder (96.7%), N/R p<br>(2) p<0.001<br>(3) p<0.05<br>(4) p<0.05<br>(5) p=0.111
**Adhesive tape vs Adhesive tape**						
15.	Liu (2011)/ China<br>Quasi	● ICU<br>● Age: 18-77	● Durapore tape (n=318)<br>● Adhesive tape (n=320)	(1) Displacement<br>(2) UE<br>(3) Pressure injury		(1) Durapore (26.7%) vs Adhesive (8.8%), p<0.05<br>(2) Durapore (5.7%) vs Adhesive (0.9%), p<0.05<br>(3) Durapore (6.3%) vs Adhesive (0%), p<0.05
16.	Chen (2013)/ China<br>Quasi	● Medical ICU<br>● Age: 71.04±7.32<br>● Excl.: oral diseases	● Elastic tape (n=48)<br>● Adhesive tape (n=52)	(1) Displacement<br>(2) UE<br>(3) Oral mucosa injury<br>(4) Oral hygiene		(1) Elastic (6.3%) vs Adhesive (46.2%), p<0.05<br>(2) Elastic (4.2%) vs Adhesive (19.2%), p<0.05<br>(3) Elastic (6.3%) vs Adhesive (38.5%), p<0.05<br>(4) Elastic (12.5%) vs Adhesive (94.2%), p<0.05
17.	Gou et al. (2015)/ China<br>Quasi	● ICU<br>● Age: 25-86	● Around neck (n=45)<br>● Crossed (n=40)	(1) Displacement<br>(2) UE		(1) Neck (17.8%) vs Crossed (50%), N/R P<br>(2) Neck (0%) vs Crossed (5%), N/R P
18.	Zeng et al. (2016)/Singapore<br>RCT	● ICU<br>● Age: 21-83	● Acrylate Adhesive tape (n=30)<br>● Silicone Adhesive tape (n=30)	(1) Skin erythema, edema (scale, event)<br>(2) Denudation (scale, event)	1st postoperative day	(1) Acrylate (50%) vs Silicone (33%), p=0.147<br>(2) Acrylate (1.3%) vs Silicone (0%), p=0.026<br>(3) p=0.001

	Author(yr)/Country<br>Design	Sample Characters	Intervention	Outcome measure	Measurement/time	Comparison Between Groups
				(3) Skin injury evaluate by anesthesiologist(event)<br>(4) Patient satisfaction (eyelids, face) Likert		(4) p<0.001, <0.001
19.	Fang, Chen (2016)/ China<br>Quasi	● Medical ICU<br>● Age: 63.62±17.71	● E-shape Adhesive tape (n=42)<br>● Y-shape Adhesive tape (n=44)	(1) Displacement<br>(2) UE<br>(3) Oral mucosa injury<br>(4) Patient satisfaction (Faces Pain Rating Scale)	(3) Oral care Twice daily<br>(4) Twice daily	(1) E (9.5%) vs Y (36.4%), p=0.03<br>(2) E (0%) vs Y (6.8%), p=0.24<br>(3) E (7.1%) vs Y (29.5%), p<0.01<br>(4) p<0.01
20.	Chen et al. (2017)/ China<br>Quasi	● ICU<br>● Age: 58-71	● Modified tape (n=45)<br>● Adhesive tape (n=45)	(1) Displacement<br>(2) Pressure injury<br>(3) Pressure injury at neck<br>(4) Patient satisfaction (VAS 0-10)<br>(5) Nurse satisfaction<br>(6) Time<br>(7) Change times	Oral care TID	(1) Modified (0%) vs Adhesive (20%), p=0.003<br>(2) Modified (0%) vs Adhesive (100%), p=0.000<br>(3) Modified (4.4%) vs Adhesive (84.4%), p=0.000<br>(4) Modified (71.1%) vs Adhesive (28.9%), p=0.000<br>(5) Modified (100%) vs Adhesive (33.3%), p=0.000<br>(6) Modified (10.31±1.47) vs Adhesive (15.78±1.89), p<0.01<br>(7) Modified (1.89±0.77) vs Adhesive (3.86±1.18), p<0.01
21.	Chen et al. (2020)/ China<br>Quasi	● ICU<br>● Age: 61.9±10.71	● Elastic tape (n=50)<br>● Durapore tape (n=50)	(1) Displacement<br>(2) Pressure injury<br>(3) Oral mucosa injury<br>(4) Oral hygiene<br>(5) Patient satisfaction<br>(6) Time		(1) Elastic(46%) vs Durapore(70%), p=0.03<br>(2) Elastic(0%) vs Durapore(10%), p=0.001<br>(3) Elastic(12%) vs Durapore(30%), p=0.027<br>(4) Elastic(20%) vs Durapore(42%), p=0.017<br>(5) Elastic(80%) vs Durapore(50%), p=0.001<br>(6) Elastic (15.18±2.51) vs Durapore (21.34±2.62) , p<0.05
22.	Xia (2020) / China<br>Quasi	● ICU<br>● Age: 64.83±14.21	● Durapore tape (n=28)<br>● Adhesive tape (n=25)	(1) Displacement<br>(2) Oral mucosa injury		(1) Durapore (4.6%) vs Adhesive (9.9%), p<0.01<br>(2) Durapore (3.6%) vs Adhesive (24%), p<0.05
23.	Bahadori et al. (2022) /USA<br>RCT	● ICU	● R’t: Hy-tape, L’t: Durapore(n=71)<br>● R’t: Durapore, L’t: Hy-tape(n=70)	(1) Erythema<br>(2) Oedema<br>(3) Scaling<br>(4) Tearing		(1) p=0.791<br>(2) p=0.344<br>(3) p=0.308<br>(4) >p=0.999
**Adhesive tape vs ETT holder**						
24.	Yu (2010)/ China<br>Quasi	● ICU	● ETT holder (n=20)<br>● Adhesive tape (n=20)	(1) Displacement<br>(2) UE		(1) holder (20%) vs Adhesive (85%), p<0.05<br>(2) holder (0%) vs Adhesive (5%), p<0.05
25.	Sheng et al. (2010)/ China<br>Quasi	● ICU	● ETT holder (n=20)<br>● Adhesive tape (n=20)	(1) Displacement<br>(2) Oral hygiene		(1) holder (16%) vs Adhesive (44%), p<0.01<br>(2) holder (20%) vs Adhesive (88%), p<0.001
26.	Zhen, Xu (2011)/ China<br>Quasi	● ICU	● ETT holder (n=33)<br>● Adhesive tape (n=32)	(1) Displacement<br>(2) UE<br>(3) Pressure injury		(1) holder (6%) vs Adhesive (25%), p<0.05<br>(2) holder (0%) vs Adhesive (12.5%), p<0.05<br>(3) holder (0%) vs Adhesive (15.6%), p<0.05
27.	Buckley et al.(2016)/USA<br>RCT	● ICU	● ETT holder (n=30)<br>● Tape (n=30)	(1) Displacement>1cm<br>(2) Displacement>4cm		(1) p<0.001<br>(2) p=0.004

	Author(yr)/Country Design	Sample Characters	Intervention	Outcome measure	Measurement/time	Comparison Between Groups
28.	Byun er al. (2016)/Korea<br>RCT	● ICU	● ETT holder (n=24)<br>● Tape (n=24)	(1) Changes in tracheal depth and bronchial depth<br>(2) DLT displacement (event)		(1) p=0.058, 0.132<br>(2) p=0.016
29.	Liao et al. (2016)/Taiwan<br>Quasi	● ICU	● ETT holder (n=46)<br>● Adhesive tape (n=51)	(1) Pressure injury<br>(2) Time<br>(3) Cost(daily)		(1) No mentioned p-value<br>(2) p<0.001<br>(3) p<0.001
30.	Zhang (2016) / China<br>Quasi	● ICU<br>● Prone position	● ETT holder (n=60)<br>● Adhesive tape (n=60)	(1) Displacement<br>(2) Movement (cm)<br>(3) Pressure injury<br>(4) Oral mucosa injury<br>(5) Nurse satisfaction (Likert)<br>(6) Time		(1) holder (5%) vs Adhesive (45%), p<0.05<br>(2) holder (3.1±1.7) vs Adhesive (13.1±2.1), p<0.05<br>(3) holder (5%) vs Adhesive (21.6%), p<0.05<br>(4) holder (38.3%) vs Adhesive (35%), p>0.05<br>(5) holder (4.97±0.55) vs Adhesive (2.32±0.78), p<0.05<br>(6) holder (22.6±4.12) vs Adhesive (70±11.52), p<0.05
31.	Chen et al. (2016)/ China<br>Quasi	● ICU	● ETT holder (n=18)<br>● Durapore tape (n=18)	(1) Displacement<br>(2) Pressure injury<br>(3) Oral hygiene<br>(4) Time<br>(5) Cost	Oral care<br>Twice daily	(1) holder (8.3%) vs Durapore (44.4%), p<0.001<br>(2) holder (16.7%) vs Durapore (100%), p<0.05<br>(3) holder (3.83±0.26) vs Durapore (3.7±0.33), p>0.05<br>(4) holder (52.27±1.11) vs Durapore (67.11±1.23), p<0.05<br>(5) holder (61.1±5.71) vs Durapore (70.1±3.67), p<0.05
32.	Zheng et al.(2016)/ China<br>Quasi	● Neurological ICU	● ETT holder (n=30)<br>● Adhesive tape (n=30)	(1) Displacement<br>(2) Pressure injury<br>(3) VAP<br>(4) Patient satisfaction (VAS 0-100)		(1) holder (6.7%) vs Adhesive (33.3%), p<0.05<br>(2) holder (6.7%) vs Adhesive (33.3%), p=0.01<br>(3) holder (10%) vs Adhesive (20%), p=0.472<br>(4) holder (0%) vs Adhesive (13.3%), p=0.023
33.	Yang (2017)/ China<br>Quasi	● ICU<br>● Prone position	● ETT holder (n=40)<br>● Adhesive tape (n=40)	(1) Displacement<br>(2) Movement (cm)<br>(3) Pressure injury<br>(4) Nurse satisfaction (VAS 0-10)		(1) holder (20%) vs Adhesive (80%), p<0.05<br>(2) holder (3.4±2.9) vs Adhesive (12.0±3.4), p<0.05<br>(3) holder (47.5%) vs Adhesive (60%), p>0.05<br>(4) holder (6.7±2.4) vs Adhesive (3.1±1.3), p<0.05
34.	Bai et al. (2018)/ China<br>Quasi	● ICU<br>● Prone position	● ETT holder (n=32)<br>● Adhesive tape (n=28)	(1) Displacement<br>(2) Movement (cm)<br>(3) Pressure injury<br>(4) Nurse satisfaction		(1) holder (28.1%) vs Adhesive (67.9%), p<0.05<br>(2) holder (3.1±1.2) vs Adhesive (11.8±2.5), p<0.05<br>(3) holder (6.25%) vs Adhesive (28.57%), p<0.05<br>(4) holder (7.25±1.34) vs Adhesive (5.09±1.82), p<0.05
35.	Chen, Lin (2019)/ China<br>Quasi	● ICU<br>● Prone position	● ETT holder (n=39)<br>● Adhesive tape (n=39)	(1) Movement (cm)<br>(2) Pressure injury<br>(3) Oral mucosa injury<br>(4) Nurse satisfaction<br>(5) Time		(1) holder (3.5±1.9) vs Adhesive (5.1±2.4), p=0.002<br>(2) holder (5.1%) vs Adhesive (20.5%), p=0.042<br>(3) holder (2.6%) vs Adhesive (18.0%), p=0.025<br>(4) holder (94.9%) vs Adhesive (76.9%), p=0.023<br>(5) holder (28.7±5.1) vs Adhesive (32.8±6.7), p=0.003
36.	Landsperger et al. (2019)/USA<br>RCT	● MICU	● ETT holder (n=153)<br>● Adhesive tape (n=145)	(1) Displacement<br>(2) UE<br>(3) Pressure injury		(1) p=0.03<br>(2) p=0.957<br>(3) p=0.05

	Author(yr)/Country<br>Design	Sample Characters	Intervention	Outcome measure	Measurement/time	Comparison Between Groups
				(4) Oral mucosa injury<br>(5) ETT repositioned (event)<br>(6) VAP (event)<br>(7) ICU mortality (event)		(4) p=0.61<br>(5) p<0.001<br>(6) N/A<br>(7) p=0.83
37.	Cao, Wei (2021)/ China<br>Quasi	● ICU<br>●	● ETT holder (n=44)<br>● Adhesive tape (n=43)	(1) Displacement<br>(2) UE<br>(3) Movement (cm)<br>(4) Pressure injury<br>(5) Oral mucosa injury<br>(6) Time		(1) holder (41.8%) vs Adhesive (20.5%), p=0.032<br>(2) holder (2.27%) vs Adhesive (18.6%), p=0.032<br>(3) holder (8.56±1.24) vs Adhesive (12.67±2.23), p<0.001<br>(4) holder (4.55%) vs Adhesive (20.9%), p=0.022<br>(5) holder (2.27%) vs Adhesive (18.6%), p=0.032<br>(6) holder (28.65±4.85) vs Adhesive (33.12±5.34), p<0.001
38.	Yanık et al. (2022)/Turkey<br>Quasi	● CVS ICU<br>●	● ETT holder (n=43)<br>● Plaster tape (n=43)	(1) SBP<br>(2) DBP<br>(3) HR<br>(4) SpO2	*all have 5 time-point (before, during, after, after 5mins, after 15mins)	(1) p=0.09, 0.18, 0.36, 0.52, 0.03<br>(2) p=0.08, 0.00, 0.00, 0.28, 0.12<br>(3) p=0.87, 0.72, 0.87, 0.75, 0.54<br>(4) p=0.41, 0.00, 0.03, 0.00, 0.18
**ETT holder vs ETT holder**						
39.	Kuniavsky et al. (2020)/<br>Israel<br>Quasi	● ICU	● Cloth tape ETTF (n=78)<br>● ETT holder (n=77)	(1) Pressure injury<br>(2) Hospital days until PI formation<br>(3) Ventilation days Until PI formation		(1) p<0.01<br>(2) p<0.01<br>(3) p<0.01
**Triple comparison & others**						
40.	He et al. (2010)/ China<br>Quasi	● ICU	● Transpore tape (n=30)<br>● Durapore tape (n=30)<br>● Twill (n=30)	(1) Displacement<br>(2) UE		(1) Transpore (66.7%) vs Durapore (53.3%) vs Twill (20%), p<0.01<br>(2) Transpore (23.3%) vs Durapore (13.3%) vs Twill (6.7%), p<0.01<br>Transpore (23.3%) vs Durapore (13.3%), p>0.05<br>Transpore (23.3%) vs Twill (6.7%), p<0.05<br>Durapore (13.3%) vs Twill (6.7%), p<0.05
41.	Mohammed, Hassan (2015)/<br>Egypt<br>RCT	● ICU	● Twill (n=30)<br>● Adhesive tape (n=30)<br>● Simple bow (n=30)	(1) Displacement (slippage scale)<br>(2) EJVP measure<br>(3) Oral mucosa injury<br>(4) Pressure injury<br>(5) Time<br>(6) Pain intensity<br>(7) Patient satisfaction (PSQ)	(1)(2)<br>15, 30, 60, 120 mins<br>(3)(4)<br>2, 6, 12, 24 hrs<br>(5)<br>5, 10, 20 mins	(1) p=0.16, 0.85, 0.64, 0.02<br>(2) p=0.32, 0.02, 0.04, 0.001<br>(3) p=0.33, 0.74, 0.14, 0.07<br>(4) p=0.61, 0.01, 0.04, 0.02<br>(5) p<0.001, <0.001<br>(6) p<0.001, <0.001, <0.001<br>(7) p=0.003
42.	Liang (2015)/ China<br>Quasi	● ICU	● ETT holder (n=30)	(1) Complication Events<br>(2) UE		(1) holder (0%) vs syringe (26.7%) vs bite (13.3%), p<0.05<br>(2) holder (3.3%) vs syringe (30%) vs bite (23.3%), p<0.05

	Author(yr)/Country Design	Sample Characters	Intervention	Outcome measure	Measurement/time	Comparison Between Groups
			● Adhesive tape with syringe (n=30)<br>● Adhesive tape with bite (n=30)			
43.	Hsieh et al. (2017)/ China<br>Quasi	● ICU	● Elastic tape (n=40)<br>● ETT holder (n=40)<br>● Adhesive tape (n=40)	(1) Displacement<br>(2) Oral mucosa injury<br>(3) Oral hygiene<br>(4) Patient satisfaction(VAS 0-10)<br>(5) Time		(1) Elastic (27.5%) vs holder(65%) vs Adhesive (75%), p=0.000<br>(2) Elastic (2.5%) vs holder(22.5%) vs Adhesive (12.5%), p=0.026<br>(3) Elastic (12.5%) vs holder(25%) vs Adhesive (70%), p=0.000<br>(4) Elastic (85%) vs holder(70%) vs Adhesive (27.5%), p=0.000<br>(5) Elastic (6±1.03) vs holder(6.57±1.82) vs Adhesive (7.46±1.53), p=0.000
44.	Sun et al. (2019)/China<br>Quasi	● ICU	● ETT holder (n=31)<br>*Walker<br>● Adhesive tape Y- & I-shaped (n=31)<br>● Alternation (n=33)<br>*Alternated daily	(1) Displacement<br>(2) Pressure injury<br>(3) Tongue injuries (event)<br>(4) Teeth loosening (event)		(1) p=0.013(among 3 groups)<br>(2) p=0.061(among 3 groups)<br>(3) p<0.001 (among 3 groups)<br>(4) p<0.001 (among 3 groups)
45.	Zhang et al. (2019)/ China<br>Quasi	● ER, ICU	● Silicone ETT holder (n=50)<br>● Adhesive tape (n=50)<br>● ETT holder (n=50)	(1) Displacement<br>(2) UE<br>(3) Facial injury<br>(4) Oral hygiene<br>(5) Time	Q8H<br>Oral care BID	(1) Silicone (40%) vs Adhesive (68%) vs holder (50%), p<0.05<br>Silicone (40%) vs Adhesive (68%), p=0.000<br>Silicone (40%) vs holder (50%), p=0.157<br>Adhesive (68%) vs holder (50%), p=0.011<br>(2) Silicone (0%) vs Adhesive (2%) vs holder(0%), p<0.05<br>(3) Silicone (18%) vs Adhesive (46%) vs holder(40%), p<0.05<br>Silicone (18%) vs Adhesive (46%), p<0.05<br>Silicone (18%) vs holder (40%), p<0.05<br>Adhesive (46%) vs holder (40%), p>0.05<br>(4) Silicone (54%) vs Adhesive (82%) vs holder (66%), p<0.05<br>Silicone (54%) vs Adhesive (82%), p<0.05<br>Silicone (54%) vs holder (66%), p>0.05<br>Adhesive (82%) vs holder (66%), p<0.05<br>(5) p<0.05

Notes: TFXB: Twill tape with FlexBlue bite block; VFXB: Velcro tie with FlexBlue bite block; ETT: Endotracheal tube; OAG: Oral assessment guild; PSQ: Patient satisfaction questionnaire; UE: Unplanned extubation; NPIAP tool: National Pressure Injury Advisory Panel tool; RCT: randomized controlled trial

Table S4. *Quality assessment of individual RCTs using Cochrane risk of bias 2 tool*

Study (First author, year)	Randomization process	Deviations from intended interventions	Missing outcome data	Outcome measurement	Selective reporting	Overall RoB
Mohammed & Hassan, 2015	🟡	🟡	🟡	🟢	🟢	🟡
Zeng et al., 2016	🟢	🟡	🟢	🟢	🟢	🟡
Buckley et al., 2016	🔴	🟡	🟢	🟢	🟢	🔴
Byun et al., 2016	🟢	🟡	🟢	🟢	🟢	🟡
Landsperger et al., 2019	🟢	🟡	🟢	🟢	🟢	🟡
Bahadori et al., 2022	🟢	🟡	🟢	🟢	🟢	🟡
Genc & Yildiz, 2022	🟢	🟡	🔴	🟢	🟢	🔴
Niu et al., 2023	🟢	🟡	🟢	🟢	🟢	🟡

🟢, Low risk; 🟡, Some concern; 🔴, High risk

Table S5. *Quality assessment of quasi-experimental studies using the JBI appraisal tool*

Article	Q1	Q2	Q3	Q4	Q5	Q6	Q7	Q8	Q9	Total
Levy, Griego 1993	●	●	⚠	●	N/A	●	⚠	●	●	🟡
Barnason et al. 1998	●	●	●	●	●	●	●	●	●	🟢
Clarke 1998	●	●	⚠	●	N/A	⚠	⚠	●	●	🟡
Seyedhosseini et al. 2017	●	●	●	⚠	N/A	⚠	⚠	●	●	🟡
Hampson 2018	●	●	●	⚠	N/A	●	⚠	●	●	🟢
Sun.2019	●	●	●	●	N/A	●	⚠	●	●	🟢
Yanık 2020	●	●	✗	●	●	●	●	●	●	🟡
Kuniavsky 2020	●	●	●	⚠	N/A	●	⚠	●	●	🟢
Ali .2022	●	●	●	●	N/A	●	●	●	●	🟢
Wang et al. 2009	●	●	●	⚠	N/A	⚠	⚠	●	●	🟡
Yu 2010	●	●	●	⚠	N/A	⚠	⚠	●	●	🟡
Chen et al. 2010	●	●	●	⚠	N/A	⚠	⚠	●	●	🟡
Sheng et al. 2010	●	●	●	⚠	N/A	⚠	⚠	●	●	🟡
Hsieh 2010	●	●	●	⚠	N/A	⚠	⚠	●	●	🟡
He et al. 2011	●	●	●	⚠	N/A	⚠	⚠	●	●	🟡
Zhen, Xu 2011	●	●	●	⚠	N/A	⚠	⚠	●	●	🟡
Liu 2011	●	●	●	⚠	N/A	⚠	⚠	●	●	🟡
Chen 2013	●	●	●	⚠	N/A	⚠	⚠	●	●	🟡
Liang 2015	●	●	●	⚠	N/A	⚠	⚠	●	●	🟡
Gou et al. 2015	●	●	●	⚠	N/A	⚠	⚠	●	●	🟡
Fang, Chen 2016	●	●	●	⚠	N/A	⚠	⚠	●	●	🟡
Liao et al. 2016	●	●	●	⚠	N/A	●	●	●	●	🟢
Zhang 2016	●	●	●	⚠	N/A	⚠	⚠	●	●	🟡
Chen et al. 2016	●	●	●	⚠	N/A	⚠	⚠	●	●	🟡
Zheng et al. 2016	●	●	●	⚠	N/A	⚠	⚠	●	●	🟡
Lee 2016	●	●	●	⚠	N/A	⚠	⚠	●	●	🟡
Hsieh et al. 2017	●	●	●	⚠	N/A	⚠	⚠	●	●	🟡
Hsu 2017	●	●	●	⚠	N/A	⚠	⚠	●	●	🟡
Chen et al. 2017	●	●	●	⚠	N/A	⚠	⚠	●	●	🟡
Yang 2017	●	●	●	⚠	N/A	⚠	⚠	●	●	🟡
Bai et al. 2018	●	●	●	⚠	N/A	⚠	⚠	●	●	🟡
Zhang et al. 2019	●	●	●	⚠	N/A	⚠	⚠	●	●	🟡
Chen, Lin 2019	●	●	●	⚠	N/A	⚠	⚠	●	●	🟡
Chen et al. 2020	●	●	●	⚠	N/A	⚠	⚠	●	●	🟡
Xia 2020	●	●	●	⚠	N/A	⚠	⚠	●	●	🟡
Cao, Wei 2021	●	●	●	⚠	N/A	⚠	⚠	●	●	🟡
Su 2021	●	●	●	⚠	N/A	⚠	⚠	●	●	🟡

Q1: Temporality; Q2: Control group; Q3: Baseline similarity; Q4: Co-intervention, Q5: Pre-post measure; Q6: Measurement consistency; Q7: Measurement validity; Q8: Follow-up; Q9: Statistical analysis

● , Yes; ⚠ , Unclear; ✗ , No.

Table S6. *Inconsistency test results for tube displacement in the network meta-analysis*

Comparison	Studies	NMA	Direct	Indirect	Diff.	95CIL	95CIU	*p* value
Adhesive：ETT Holder	12	1.92	1.92	NA	NA	NA	NA	NA
Adhesive：Twill	6	1.39	1.39	NA	NA	NA	NA	NA
ETT Holder：Twill	0	-0.53	NA	-0.53	NA	NA	NA	NA

Diff: Difference; 95CIL: lower limit of 95% confidence interval; 95CIU: upper limit of 95% confidence interval; NMA: network meta-analysis

Table S7. *Inconsistency test results for pressure injury in the network meta-analysis*

Comparison	Studies	NMA	Direct	Indirect	Diff.	95CIL	95CIU	*p* value
Adhesive：ETT Holder	12	1.12	1.15	0.72	0.43	-2.62	3.49	0.78
Adhesive：Twill	3	1.28	1.17	1.60	-0.43	-3.49	2.62	0.78
ETT Holder：Twill	2	0.16	0.45	0.02	0.43	-2.62	3.49	0.78

Diff: Difference; 95CIL: lower limit of 95% confidence interval; 95CIU: upper limit of 95% confidence interval; NMA: network meta-analysis

Figure S1. *Summary of quality assessment of studies included in the network meta-analysis using Cochrane risk of bias 2 tool*

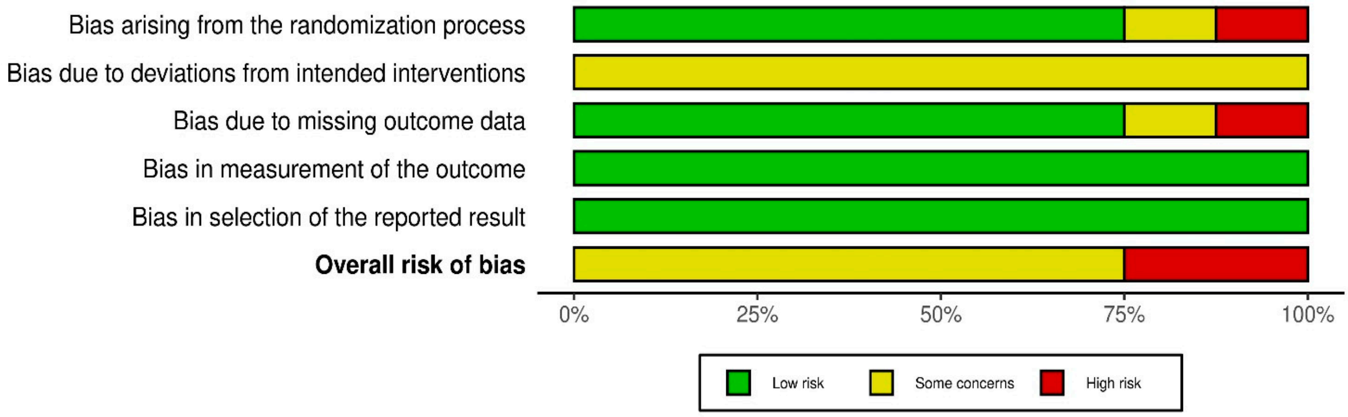

Figure S2. *Forest plots of leave-one-out sensitivity analysis for tube displacement.*

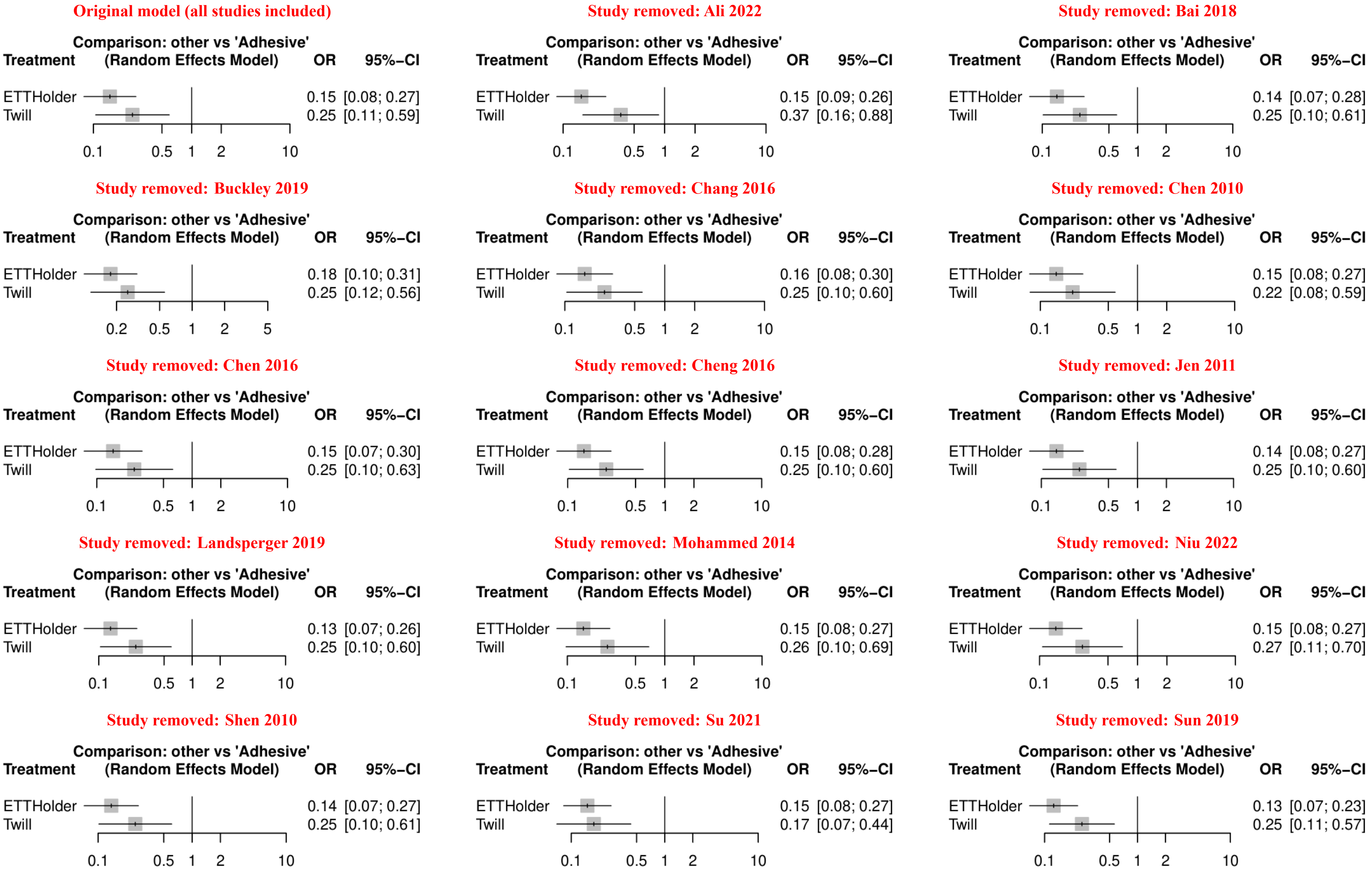

Study removed: Wang 2009

Study removed: Yang 2017

Study removed: Yu 2010

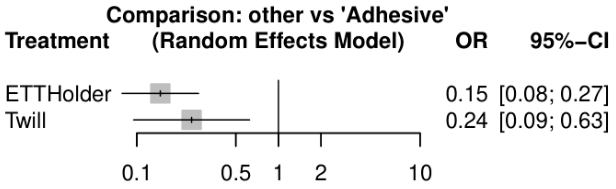

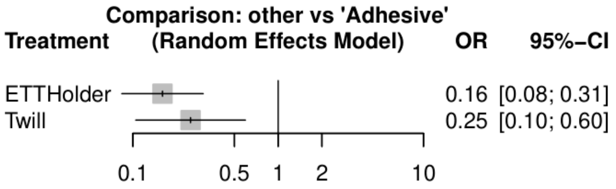

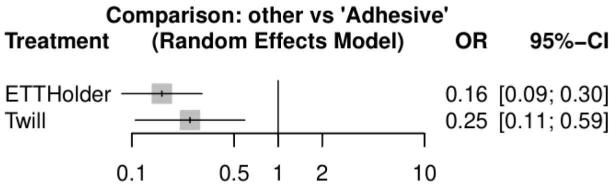

Study removed: Zao 2021

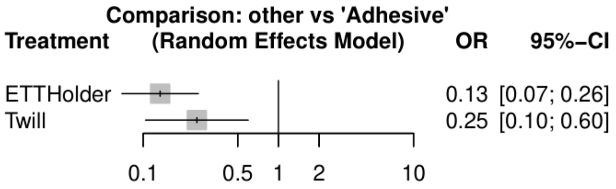

Figure S3. *Forest plots of leave-one-out sensitivity analysis for pressure injury*

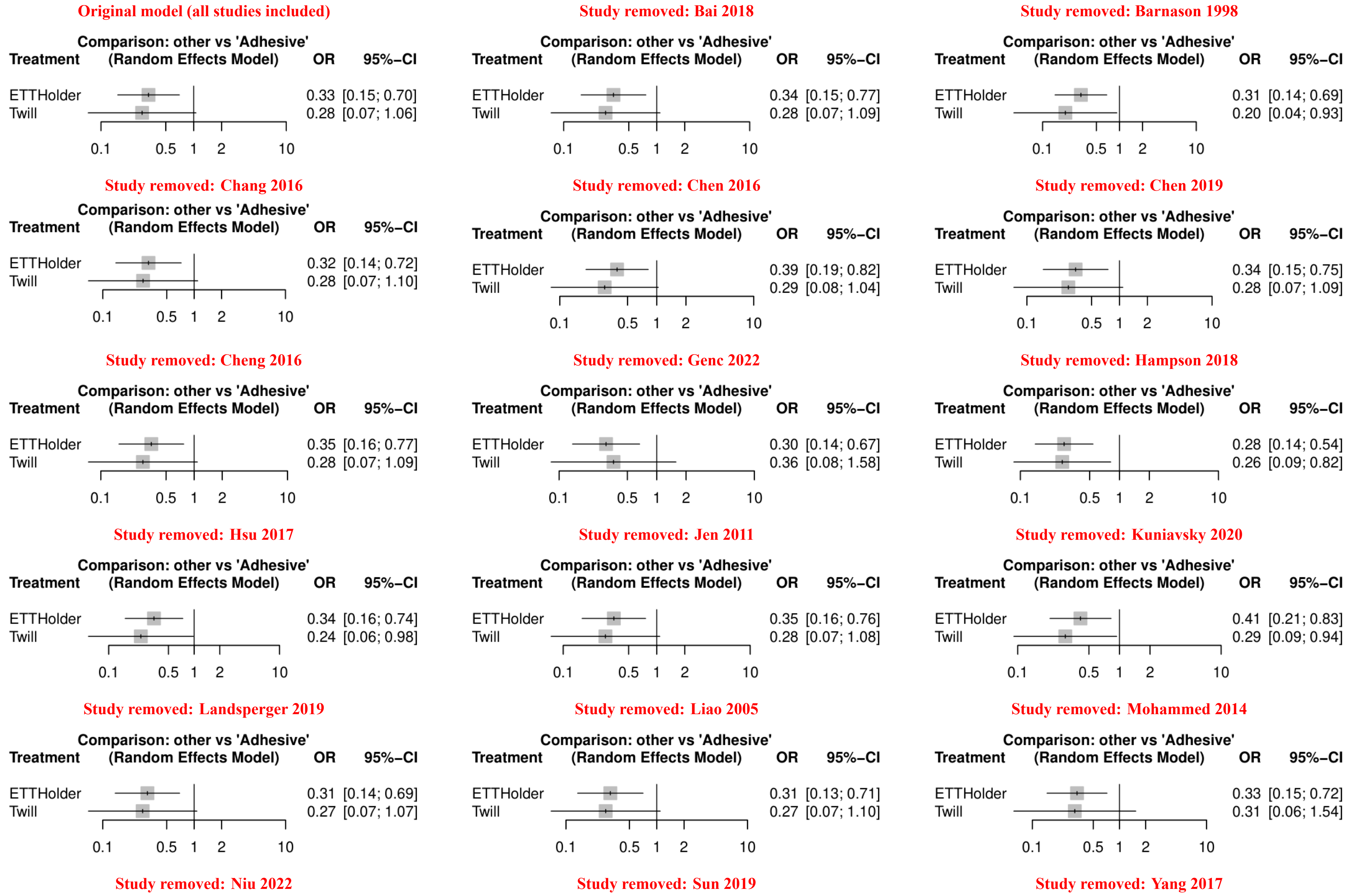

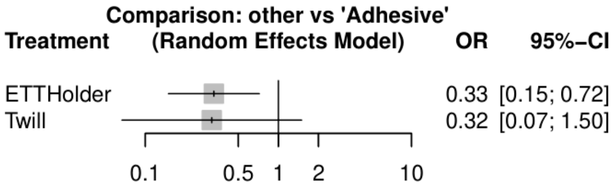
Comparison: other vs 'Adhesive'
Treatment
(Random Effects Model)
OR
95%−CI
ETTHolder
0.33 [0.15; 0.72]
Twill
0.32 [0.07; 1.50]
0.1
0.5
1
2
10

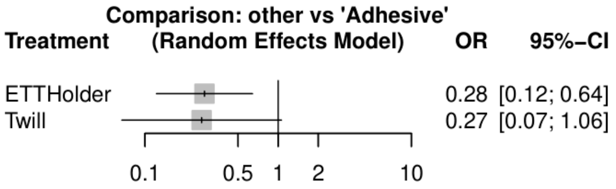
Comparison: other vs 'Adhesive'
Treatment
(Random Effects Model)
OR
95%−CI
ETTHolder
0.28 [0.12; 0.64]
Twill
0.27 [0.07; 1.06]
0.1
0.5
1
2
10

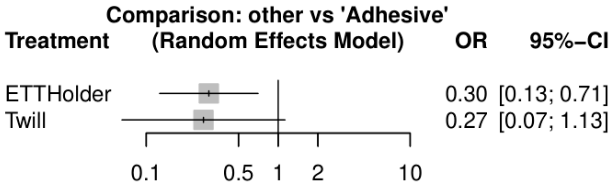
Comparison: other vs 'Adhesive'
Treatment
(Random Effects Model)
OR
95%−CI
ETTHolder
0.30 [0.13; 0.71]
Twill
0.27 [0.07; 1.13]
0.1
0.5
1
2
10

Figure S4. *Funnel plot: Adhesive tape vs ETT holder in displacement event*

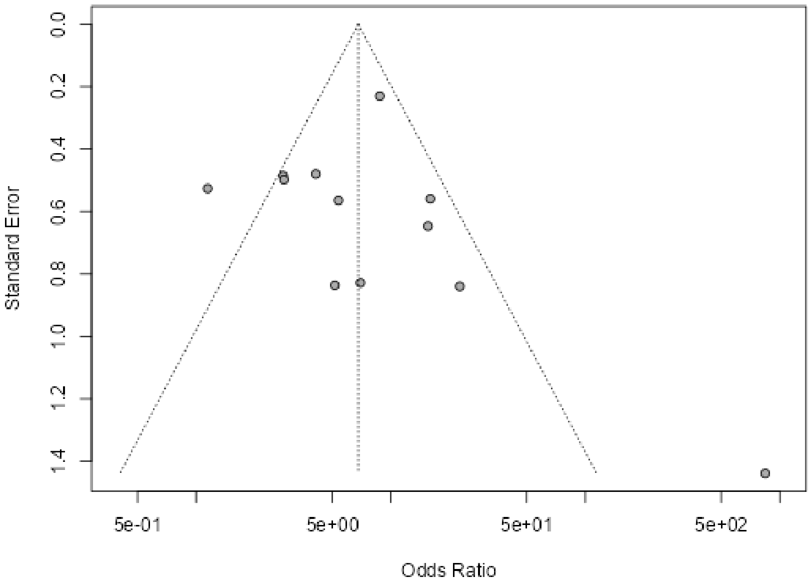

Figure S5. *Funnel plot: Adhesive tape vs ETT holder in pressure injury event*

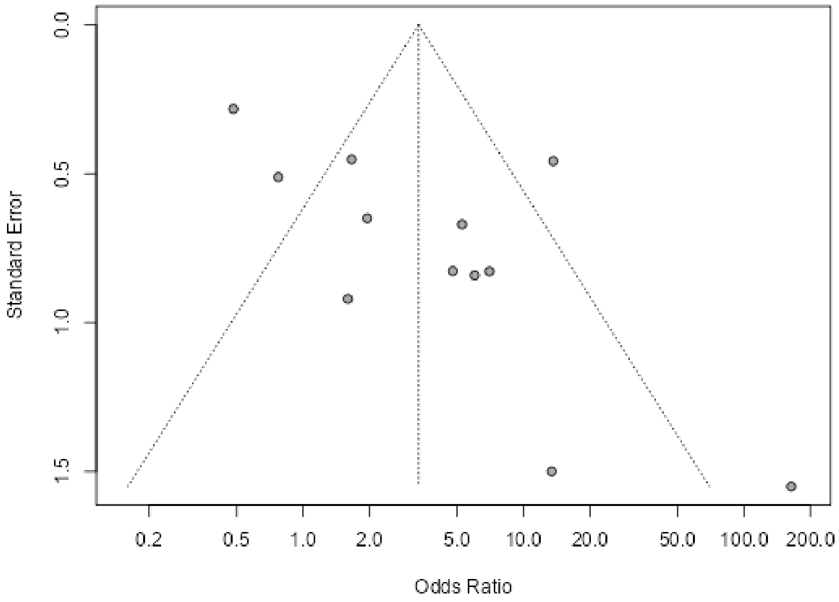

Supplement: Supplementary file 1 [file nursrep-16-00317-s001.zip › nursrep-4439936-supplementary.pdf]
